# Shaping Hierarchical Porosity Beads from Metal–Organic Framework Powders Using Freeze Spherification

**DOI:** 10.1021/acs.chemmater.5c00901

**Published:** 2025-10-30

**Authors:** Adrián Quindimil, Roberto Fernández de Luis, Stefan Wuttke, Jonas Gurauskis

**Affiliations:** † Instituto de Nanociencia y Materiales de Aragón (INMA), Consejo Superior de Investigaciones Científicas (CSIC)−Universidad de Zaragoza (UNIZAR), Calle Mariano Esquillor 15, CIRCE Building, Zaragoza 50018, Spain; ‡ BCMaterials, Basque Center for Materials Applications and Nanostructures, UPV/EHU Science Park, Leioa 48940, Spain; § Academic Centre for Materials and Nanotechnology, 49811 AGH University of Krakow, Krakow 30-059, Poland; ∥ Fundación Agencia Aragonesa Para la Investigación y el Desarrollo (ARAID), Avenida de Ranillas 1D, Zaragoza 50018, Spain

## Abstract

Microporous powdered Metal–Organic Frameworks (MOFs) shaped by conventional mechanical densification methods (e.g., pelletization, granulation) present internal diffusion limitations and pressure drops that limit their technological leap to industrial applications. Therefore, the ongoing search for novel shaping methods that could manufacture controlled hierarchical porous structures with enhanced diffusion, high surface area, crystallinity, and mechanical robustness is needed. In this work, a freeze spherification method is developed to shape MOF-808 powder into spherical beads with hierarchical micro/macro porosity. The optimum spherical bead composition, in terms of suspension solids loading (10, 20, or 30 vol %) and hydroxypropyl methylcellulose (HPMC) binder content (30, 25, and 20 wt %), is determined by considering the bulk density, MOF surface accessibility, and the formation of a highly permeable porous structure in the final beads. The MOF-808 in powder and spherical bead forms is characterized by PXRD, IR, TGA, SEM, N_2_ physisorption, and mercury porosimetry. The analyses confirm the formation of hierarchically ordered porosity with varying pore sizes, morphologies, and MOF coating degrees, which depend on both suspension solids loading and binder content. The combination of MOF-808 with the HPMC binder does not affect its crystallinity, but a reduction of the binder content of 30 vol % beads from 30 to 25 wt % is key to retain 86% of the MOF-808 surface area . Noteworthy, the beads processed under optimum conditions exhibit sufficient mechanical integrity and superior accessibility, highlighting freeze spherification as a promising shaping method to bring powdered MOF-based catalysts and adsorbents to real-world applications.

## Graphical abstract

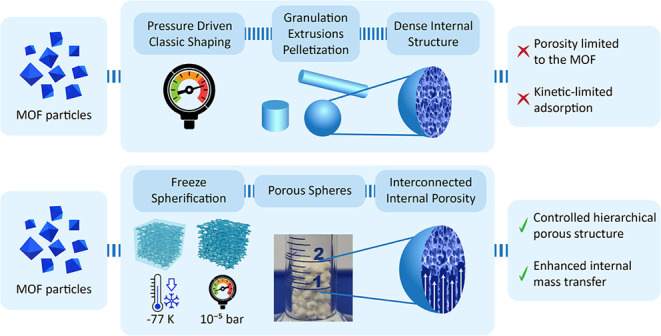

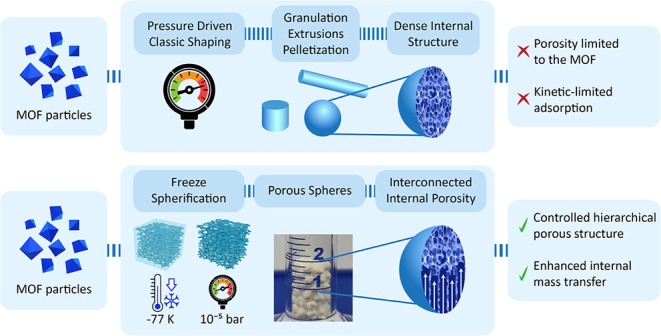

## 1 Introduction

Metal–Organic Frameworks (MOFs) are well-known porous and crystalline sorbents with high specific surface areas (1000–10,000 m^2^/g), easily tunable porosity (pore diameter = 3–20 Å), and physicochemical properties (achieved through presynthetic functionalization or postsynthetic encoding). MOFs are assembled from inorganic structural building units (transition metal ions or clusters) connected by organic linkers, forming highly ordered porous structures. The geometry and connectivity of both components allow guiding the design of MOFs toward certain topologies with given porosity metrics (i.e., surface area, pore volume, pore window).^1,2^ In addition, the frameworks offer multiple opportunities to encode them with specific functions during and after their synthesis. All in all, due to their versatility in tuning the chemistry and metrics of their pore space, the figures of merit of MOFs have become viable competitors to classical high-performance sorbents, such as zeolites or activated carbons, for the adsorption, storage, or separation of various gas and vapor molecules.^2,3^

One of the main challenges limiting the widespread application of MOFs is that they are synthesized in the form of fine polycrystalline powders that are hardly processable. In industry, the powdered form of a material is usually incompatible with the majority of continuous flow adsorption or catalysis processes, as powders normally cause clogging and huge pressure drops throughout the packed bed, especially when operating at high gas flow rates.^4,5^ In order to avoid such operational problems, a great deal of research effort has been devoted to shaping MOF powders into macroscopic pellets or beads. Among these traditional shaping methods, pelletization, granulation, and extrusion are the most frequently reported techniques.^1^ Pelletization relies on the mechanical densification of MOF powders enclosed in a mold by mechanical compression, resulting in the formation of uniform cylindrical particles.^6,7^ Granulation is another mechanical densification method that results in particles with more heterogeneous (quasi-spherical) shapes and sizes.^3^ Generally, the granules can be formed by grinding and sieving pellets (dry agglomeration) or by powder agglomeration (wet granulation). In the latter case, the addition of an inorganic (e.g., SiO_2_ and mesoporous Al_2_O_3_) or organic binder (e.g., cellulose and PVA) is needed to shape and mechanically stabilize the granules. On the other hand, the introduction of a binder leads to a decrease in textural properties due to pore blocking.^8,9^ Last but not least, extrusion is the preferred shaping method in the industry, as it enables the processing of a higher volume of adsorbent within a given time frame. It involves the formulation of a paste and its subsequent conformation as an extrudate by passing it through a die of various shapes, followed by cutting the extrudate into pieces of the same size. It is important to note that packed beds composed of pellets, beads, granules, and extrudates prepared by traditional shaping still exhibit: (i) considerable pressure drops when working at high flow rates and (ii) mass transfer limitations associated with gas diffusion into or through the pellets or granules. Overall, these methods often result in limitations for gas molecules to contact the entire active surface area of the sorbents, leading to a significant reduction in efficiency and oversizing of the packed beds.

In order to improve reactant diffusion and minimize pressure drop, the development of advanced adsorbent shaping methods is highly desirable.^4^ Recently, gelation and freeze casting, or ice-templating techniques, have emerged as promising shaping methods.^10,11^ Both methods aim to create hierarchical porosity inside the macro- to nanometer-scale structure of the final shaped sorbent forms but differ in macropore formation mechanisms and in the achievable MOF loading, which ultimately influences the bulk density. Sol–gel casting, which has been widely applied to shape MOF materials,^10,12–17^ involves the preparation of a colloidal suspension (i.e., sol) followed by its gelation through a cross-linking agent. During gelation, the colloids are linked together to form chains that are organized in a 3D interconnected network, adopting the macroscopic shape of the vessel. The interconnected pore structure is created by removing the solvent entrapped within the gel by freeze or supercritical drying.^13^ When the shape of the initial gel is fully or partially retained after drying, it is usually termed an aerogel. In the context of MOFs, aerogels can be composed of pure MOF^12^ or MOF-composites.^10,13^ In the latter, MOF particles have already been immobilized in graphene,^14^ silica,^15^ cellulose,^16^ or wood^17^ aerogel scaffolds either before or after aerogel formation. MOF or MOF-composite aerogels present high specific surface area, macroporosity, and active site accessibility, which make them interesting candidates for liquid adsorption applications, such as dye removal and wastewater treatment. However, they generally present very low bulk density or too high porosity, leading to low surface-to-volume ratios, which is not desirable for continuous gas adsorption and catalysis applications.^18^ Besides, while sol–gel prepared aerogels offer more versatile and customizable properties, they still exhibit limited scalability, density, and mechanical stability.^10,13^

In contrast, freeze casting is presented as a simpler, more scalable, and viable shaping method for preparing monoliths and/or beads. This method requires the preparation of a suspension containing the adsorbent or catalyst and a binder, followed by subsequent freezing. During freezing, ice crystal fronts are formed, generating forces that push the particles apart while simultaneously fostering their cohesion. In this case, the interconnected porous structure is established by sublimation or freeze-drying of these ice crystals, which act as porous templates.^19^ Notably, the porosity and porous structure can be tuned by varying parameters that affect the ice crystal growth and morphology, such as freezing rate, freezing agent, viscosity, particle size, and solids loading.^20^ Furthermore, note that its soft shaping conditions present an advantage over classical shaping or sol–gel methods.

While freeze granulation has been widely employed in the literature to shape metal oxides, ceramics, metals, or polymers,^11,21–23^ there are few works that explore the processing of MOFs by freeze casting and/or ice templating.^24–28^ Ahmed et al.^24^ were the first to apply the freeze-casting approach to prepare a binder-free HKUST-1 monolith with aligned bimodal macroporosity and intrinsic microporosity by directly freeze casting a DMSO solution containing MOF precursors. However, in many cases, the in situ synthesis of MOFs under freeze-cast conditions is not feasible, requiring the use of a binder material. In following works, polymeric binders, such as chitosan,^25^ PSS^26^, and PVA,^27^ were employed, revealing that binders generally cause pore blocking of MOFs. In this context, Hastürk et al.^28^ studied the effect of different hydrophilic polymeric binders on the textural properties of ice-templated MIL-160­(Al) and MIL-101­(Cr) monolith MOFs for water adsorption applications. They concluded that high molecular weight binders (e.g., PVP) were more effective since they minimize pore blocking and preserve textural properties, leading to higher water uptake values. However, these studies did not explore the use of cellulose, which has gained attention as a binder.^22,29,30^ This biopolymer stands out due to its ability to form interconnected networks via hydrogen bonding, enhancing interfacial adhesion, mechanical strength, and flexibility. Besides, unlike other synthetic binders, cellulose is biodegradable and highly soluble in aqueous systems, which makes it very compatible for freeze-casting process.

Although previous studies have focused on shaping MOFs into monoliths, there has been no exploration of freeze casting for MOF bead formation. To address this gap, in this work, we develop and optimize a processing method, termed freeze spherification, to shape MOF materials into spherical beads with hierarchical porosity, based on droplet technology and a freeze-casting approach. We systematically optimize the freeze spherification method, varying key freeze-casting parameters, such as total solids (MOF and binder) loading, concentration of the initial suspension, and binder content, which play a crucial role in determining the final hierarchical porous structure and bulk density of the beads. As a proof-of-concept to demonstrate the feasibility of our novel freeze spherification method, we selected MOF-808 zirconium trimesate due to its intrinsic microporosity, the abundance of uncoordinated positions that open the space for its postsynthetic functionalization before or after freeze spherification shaping, and, last but not least, the variety of possible applications of the MOF-808 beads, including gas adsorption, separation, or catalysis, which could benefit from both the intrinsic surface area of the MOF particles as well as the external interconnected porosity. Additionally, once optimized, we further demonstrate the versatility of this method by successfully shaping other MOFs, including a mesoporous UiO-66-NH_2_ and microporous MIL-100­(Fe).

## 2 Experimental Section

### 2.1 Synthesis of MOF-808 Powder

MOF-808 was synthesized by a hydrothermal method, using zirconium chloride (ZrCl_4_) as the metal source and trimesic acid (H_3_BTC) as the organic linker.^31^ First, stoichiometric amounts of ZrCl_4_ (1.86 g, 7.98 mmol) and H_3_BTC (0.56 g, 2.66 mmol) precursors (ZrCl_4_/BTC molar ratio = 3) were dissolved separately in 40 mL of 50 vol % acetic acid/water solutions. Then, the ZrCl_4_ solution was added dropwise to the trimesic acid solution. Subsequently, the mixture was sealed in in a Pyrex autoclave under magnetic stirring at 250 rpm and kept heated in an oil bath at 110 °C for a period of 24 h. After the hydrothermal reaction, the material was recovered by centrifugation, washed two times with water and two other times with ethanol. Finally, the sample was dried under vacuum at 40 °C in two steps: first, for 4 h at 100 mbar to gradually evaporate the remaining solvent, followed by 4 h at 10 mbar to ensure complete drying.

### 2.2 Preparation of MOF Beads by Freeze Spherification

Porous MOF-808 beads with increasing solids volume fraction were prepared by the freeze spherification method (Scheme S1), varying the total solids (MOF and binder) loading of the initial suspension (10, 20, or 30 vol %). Commercial hydroxypropyl methylcellulose (HPMC, MethoCel K3 Premium LV) from *ChemPoint* was employed as the binder, and water was used as the solvent. In all cases, first, the aqueous suspension was prepared by dispersing the required amount of MOF-808 powder into the HPMC/H_2_O solution under vigorous stirring (1000 rpm) for 1 h. The suspension was then added dropwise at a 0.15 mL min^–1^ flow rate through a 1.5 mm ID tube connected to a peristaltic pump into a N_2_ liquid bath at 77 K, where droplets rapidly froze after floating briefly (5 s) on the liquid N_2_ surface and were then collected in the form of ice-templated MOF-808@HPMC beads. Finally, the beads were freeze-dried in a *Telstar* lyophilizer (−42 °C, 0.5 mbar, 24 h), allowing sublimation of ice dendrites and the formation of a 3D interconnected macropore network. MOF-808 beads are denoted as *x*M@C-*y*, where *x* represents the solids loading (10, 20, or 30 vol %) of the initial suspension, M@C denotes MOF embedded in a cellulose matrix, and *y* refers to the nominal binder content (30, 25, or 20 wt %). In total, 5 batches of MOF-808 beads were prepared, and the label, nominal composition, average mass, solids fraction, and bulk density are summarized in Table 1.

**Table 1:** Label, Nominal Composition, Average Mass, Solids Fraction, and Bulk Density of the Prepared MOF-808 Beads

Sample	SL^a^ (vol.%)	BC^b^ (wt.%)	SF^c^ (vol.%)	Avg. bead mass (mg)	Bulk density^d^,^e^ (g cm^–3^)
10M@C-30	10	30	18	1.5	0.058
20M@C-30	20	30	28	3.6	0.161
30M@C-30	30	30	41	5.4	0.272
30M@C-25	30	25	33	5.2	0.222
30M@C-20	30	20	n.a.	5.3	0.250

a Nominal solids loading (SL) of the initial MOF/HPMC aqueous suspension.

b Nominal binder content (BC) of the beads.

c Solids fraction of the beads, calculated as 100 – ε (porosity), excluding micropores.

d Measured by weighing a graduated test tube containing 5 cm^3^ of sample.

e The bulk density of MOF-808 powder is 0.615 g cm^–3^.

### 2.3 Characterization Techniques

Optical microscopy (OM). The OM images were collected using a *ZEISS SteREO Discovery.V8* microscope equipped with a PlanApo S apochromatic lens, which provides an 8:1 zoom magnification range, and illumination and contrast methods based on either cold or LED light. The brightfield OM images of the MOF-808 beads were collected employing cold light and through an Axiocam 105 color camera with 5 megapixels resolution (2.2 μm pixel size) connected to the microscope.

X-ray diffraction (XRD). The degree of crystallinity of powder and processed MOF-808 was assessed by XRD using a *Panalytical Empyrean* X-ray diffractometer with Cu-*K*α radiation (λ = 0.154 nm) at 40 kV and 40 mA. The XRD patterns were collected from 5 to 90° 2θ with a step size of 0.0131° using a *PIXcel1D Medipix3* detector.

Thermogravimetric analysis (TGA). TG experiments were conducted on a *Mettler Toledo TGA/STDA 851e* thermobalance to evaluate the thermal stability and degradation of samples. In a typical TG experiment, around 10 mg of sample was placed in a 70 μL Al_2_O_3_ crucible and heated from 35 to 700 °C at 10 °C min^–1^ under 40 cm^3^ min^–1^ air flow. First derivatives were applied to the TG profiles to calculate the differential thermogravimetry (dTG) profiles. The MOF (*X*_MOF_) and binder (*X*_Binder_) contents of the beads (wt %, dry basis) were estimated from the TG residuals at 700 °C as

1 $$X_{\mathrm{MOF}}\left(\%\right)=\frac{R_{\mathrm{bead}}}{R_{\mathrm{powder}}}\times 100;X_{\mathrm{HPMC}}\left(\%\right)=100-X_{\mathrm{MOF}}$$

where *R*_bead_ and *R*_powder_ are the residual mass fractions at 700 °C for MOF-808 beads and the pristine MOF-808 powder, respectively.

N_2_ physisorption. Textural properties of powder and bead-shaped MOF-808 samples were determined from N_2_ adsorption–desorption isotherms at 77 K, recorded by a *Micromeritics TRISTAR 3000* apparatus. The specific surface area (*SSA*) was calculated by the Brunauer–Emmett–Teller (BET) equation, while the external surface area (*S*_ext_) and micropore volume (*V*_micro_) were calculated by the *t*-plot method. Previous to the analysis, all samples were degassed at 150 °C for 10 h under vacuum. The MOF-808 accessibility, or the percentage of accessible specific surface area, was estimated as follows:

2 $$\mathrm{Surface Accessibility}\left(\%\right)=\frac{SSA_{\mathrm{bead}}}{SSA_{\mathrm{powder}}\cdot X_{\mathrm{MOF}}}\times 100$$

where *X*_MOF_ is the MOF-808 mass fraction of beads, estimated from thermogravimetric analysis. Note that the contribution of HPMC to the bead surface area was considered negligible (*SSA*_HPMC_ < 1 m^2^ g^–1^).

Scanning Electron Microscopy (SEM). The morphology and porous structure of MOF-808 powder and ice-templated beads were studied by SEM, employing an *FEI Inspect F50* model operating at 10 kV. Both surface and cross-section micrographs of the beads were obtained and analyzed at different magnifications. To obtain a completely smooth section of MOF-808 beads, the samples were cryogenized and subsequently sectioned using a cryotome. Prior to the measurements, all samples were sputter-coated with palladium to avoid charging.

Fourier Transform Infrared (FTIR) spectroscopy. The vibrational modes of the MOF-808 powder and *x*M@C-*y* beads were studied by FTIR spectroscopy. The FTIR data were acquired in attenuated total reflection (ATR) mode using Jasco FT/IR-6100 equipment in the wavelength range between 4000 and 600 cm^–1^. 64 scans at a resolution of 4 cm^–1^ were recorded and averaged to obtain the final FTIR spectra. The background signal was recorded before the measurement of each sample and subtracted from the final data. Last but not least, the baseline was corrected following the same protocol for all the spectra. Additionally, the FTIR spectra of MOF-808 were also recorded in transmission mode after diluting the sample to 1% in KBr pellets. The pellets were employed to follow up the FTIR signature after the ex-situ heating of the sample at different temperatures.

Proton nuclear magnetic resonance (^1^H NMR). Analyses were performed using a Bruker AV500 equipped with a BBI probe and *Z*-axis gradients, operating at a frequency of 500 MHz for the proton. For this purpose, 23 mg of the sample was digested overnight in a NaOH–deuterated water solution (1 M–0.7 mL). The mixture was centrifuged at 7000 rpm, and the remaining solution was recovered carefully with a syringe, preventing the uptake of the powdered material settled at the bottom of the centrifuge tube. Afterward, the molar ratio between trimesic and acetic acid molecules was obtained for MOF-808. All processes were performed with the aid of Mestre Nova software.

## 3 Results and Discussion

### 3.1 Effect of Suspension Solids Loading

First, the PXRD fingerprint of the MOF-808 powder and the beads with different solids fractions, which were previously softly ground into powder, was quantitatively compared. As shown in Figures 1a and S1, the PXRD pattern of the MOF-808 sample presents the characteristic diffraction maxima arising from the long-range ordering of the cubic *Fd*3̅*m* structure (*a* = 35.0764 Å) of the MOF-808 material.^32–34^ This structure consists of Zr_6_ clusters coordinated to six BTC^3–^ ligands, which, in turn, are linked to three Zr clusters, forming a 3D network with tetrahedral (4.8 Å) cages and larger adamantine-shaped (18 Å) cavities.^33^

**Figure 1:**
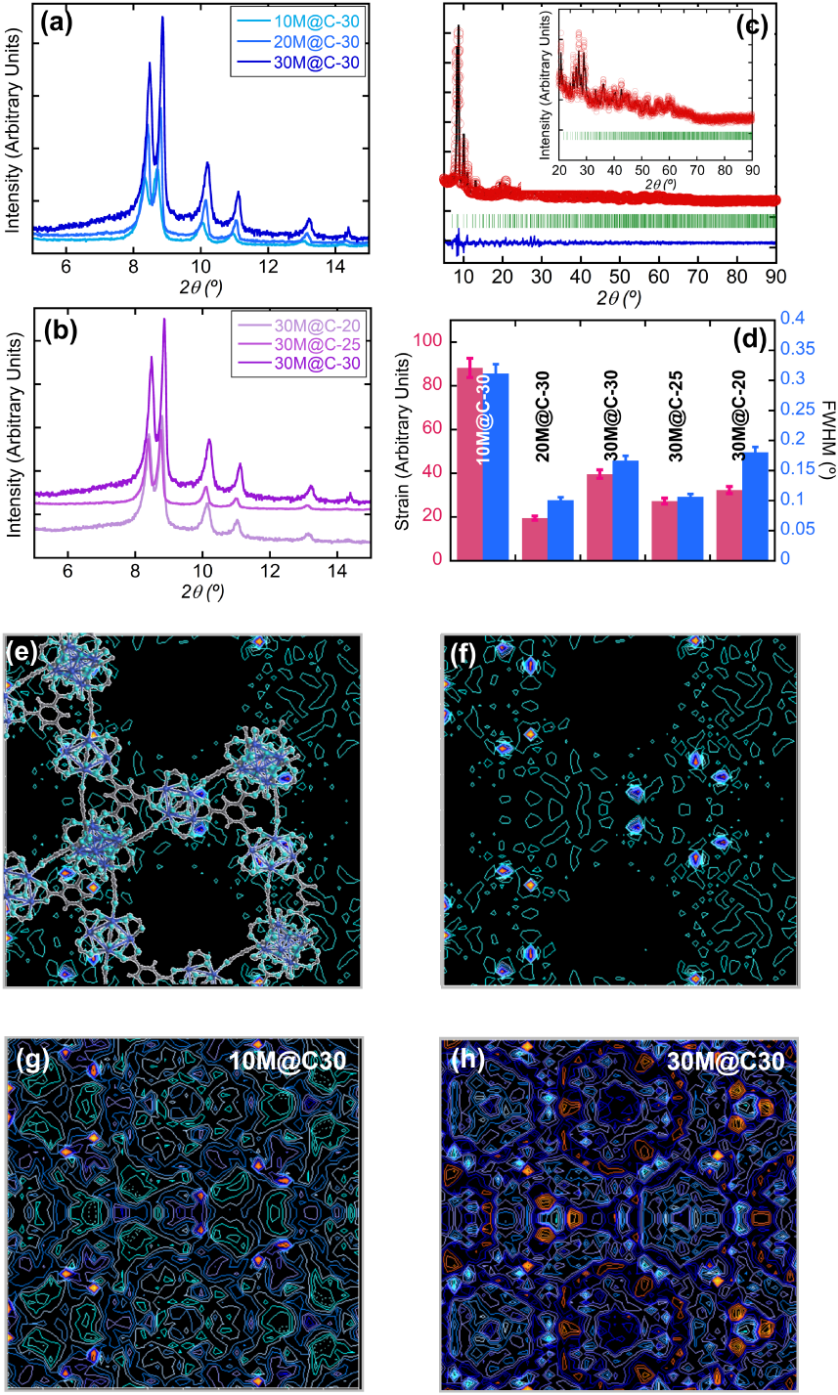
Analysis of the PXRD data of the MOF-808 powder, hydroxypropyl methylcellulose (HPMC) binder, and MOF-808 beads: (a,b) PXRD patterns, (c) final Rietveld fitting of the 10M@C-30 sample, (d) crystallographic strain and fwhm(°) values obtained from the Rietveld and (311)-maxima fittings, respectively. (e–h) Electron density maps obtained from (e–f) the observed data for 10M@C-30, and the difference between the observed and calculated data for (g) 10M@C-30 and (h) 30M@C-30.

It is important to note that a preliminary comparison of the experimental and simulated data for MOF-808 discards the presence of any impurity in the sample (Figure S1). MOF-808-shaped samples show small full-width-at-half-maximum values (fwhm), along with small contributions arising from amorphous polymeric components derived from the freeze spherification.^32^ Instead, the two broad peaks (i.e., 8.9 and 19.5̊ in 2θ) of HPMC point out that the binder is in general an amorphous solid with very limited long-range ordering (Figure S1). Two additional features are initially observed when the PXRD data of the freeze-spherificated samples are analyzed: (i) a slight increase of the background arising from the HPMC component, which depends on the shaping conditions; and (ii) a slight variation in the relative intensity of the diffraction maxima of the MOF-808 component. Said that, the intensity of the background—especially the one located close to the two broad maxima of the HPMC component—increases as the solids fraction increases, being 30M@C-30 sample the one showing the highest signal from HPMC (Figure 1a).

In order to analyze the slight variation in the relative intensities of the patterns, Rietveld refinements of the data were done starting from the structural model of MOF-808 reported by H. Furukawa et al.^35^ The solvent molecules were removed from the structure, and the refinement was done without modification of the atomic positions of the model. A resolution file—obtained from the refinement of the LaB_6_ standard—was employed to estimate the average size of the crystalline domains and the strain of the freeze-spherificated samples from the Rietveld refinement. As the same batch of MOF-808 was employed to shape all the samples, a variation in the size of the crystalline domains for MOF-808 component is discarded. Thus, we employed the value of the size of the crystalline domains obtained for MOF-808 sample (i.e., 573 ± 2 nm) in the Rietveld refinements of the freeze-spherificated samples. In fact, this value agrees with the crystal size shown in the scanning electron microscopy images. Thus, any additional broadening of the maxima was ascribed to the increase in the crystallographic strain in the MOF-808 crystals induced by the shaping process. The final fitting obtained from the Rietveld refinement of 10M@C-30 can be observed in Figure 1c. The fittings for the rest of the samples studied in this work are included in Figure S2. On the other hand, Figure 1d summarizes the value of the crystallographic strain of the freeze-spherificated samples, together with the value of the full width at half-maximum (FWHM) obtained from the profile fitting of the (311) maximum. Given the strain and FWHM values, the sample showing the lower contribution of the background signature from HPMC (i.e., 10M@C-30) is the one that exhibits the greater broadening of the diffraction maxima, and in turn, the higher crystallographic strain. Said that, it is important to mention that although the strain values are slightly different depending on the sample, in general, the MOF-808 component does not suffer severe crystallographic stress during the shaping process.

Taking a step further, we computed the residual electron density from the difference between the observed and calculated PXRD data after the Rietveld refinement. These density maps, plotted in Figure 1e–h helped us to identify whether the shaping process could give rise to an increase of the electron density in the pore space of MOF-808, arising from its partial blocking by the HPMC polymeric component. First, the electron density map of the observed data was calculated and compared with the crystal structure of MOF-808. A plot of the sum of the electron density of the different planes along the [110] crystallographic direction is observed in Figure 1e. These regions with higher electron densities correlate with the positions of the inorganic clusters and the organic linkers of the MOF-808 crystal structure. When the electron density of this framework is subtracted from the map, the residual electron density of the pore space can be observed. For example, in the case of the 10M@C-30 sample—which exhibits the lower contribution of the amorphous polymeric component in the PXRD data—a pore space relatively free of residual electron density is observed (Figure 1f). In fact, most of the residual density is concentrated near the zirconium ions of the clusters. This finding is relatively reasonable since the positions of the zirconium atoms in the initial model were not refined. In any case, the pore space, which is the region of interest in this analysis, is relatively free of positive electron density and, hence, of potential solvent or HPMC residues. In comparison, the freeze-spherificated samples showing a PXRD pattern with a significant contribution of HPMC residue (i.e., 30M@C-30) also exhibit a significant residual electron density signal in the pore space of the MOF-808 structure. For example, in the case of the 30M@C-30 sample, most of the positive residual density is concentrated inside the pore space of the supertetrahedron formed by the connection between four Zr_6_ clusters with trimesate bridges. In addition, a second positive electron density area is found surrounding the surface of the hexagonal pore windows that give access to the adamantane-like pore space. All in all, the analysis of the PXRD data indicates that these samples, showing an HPMC residue in the background signal of the patterns, also exhibit a partial blocking of their pore space, tentatively ascribed to polymeric residues.

In order to unravel how MOF-808 beads are decomposed and to assess their thermal stability, thermogravimetric analyses (TGA) of the different formulations were carried out. TG or mass loss profiles from 150 to 675 °C, along with their derivative curves (dTG) of MOF-808 powder, HPMC binder, and 20M@C-30 beads, are shown in Figure 2a, while the rest of the profiles are included in Figure S3. In general, the mass loss occurs in different steps that can be identified by dTG curves. In the case of the MOF-808 powder, the mass loss below 150 °C (Figure S3) is associated with solvent release. This initial step is followed by the loss of the acetate modulator and the dehydration of the [Zr_6_O_4_(OH)_4_]^12+^ clusters between 150 and 300 °C. Finally, the calcination of the trimesate linkers occurs above 350 °C.^34,36–38^ Note that the dTG peak at around 570 °C may be attributed to the combustion of the remaining organic compounds, leading to a pronounced final mass loss. As a result of thermal decomposition, ZrO_2_ remains, which represents 53 wt % of the mass on a dry basis. MOF-808 was further characterized by ^1^H NMR after its digestion in a NaOH–1 M deuterated solution. As expected, proton shifts recorded in the spectra (Figure S4) match the usual ones of trimesic and acetic acid components. The integration of selected signals indicates a molar ratio of 0.5 to 1.0 of trimesic acid with respect to acetic acid. Taking this information into account, the following average formula, Zr_6_(μ_3_-O)_4_(μ_3_-OH)_4_ (μ_1_-OH)_2_(μ_1_-H_2_O)_2_(C_9_H_3_O_6_)_2_(C_2_H_3_O_2_)_4_, for MOF-808 can be proposed if the coordination positions in the clusters not occupied by acetate and trimesate molecules are compensated by water/hydroxyl pairs. In fact, considering this formula, a value for the residual weight of 52.6% after the calcination of the initial material can be calculated, a value that is very close to the experimental one observed by TGA.

**Figure 2:**
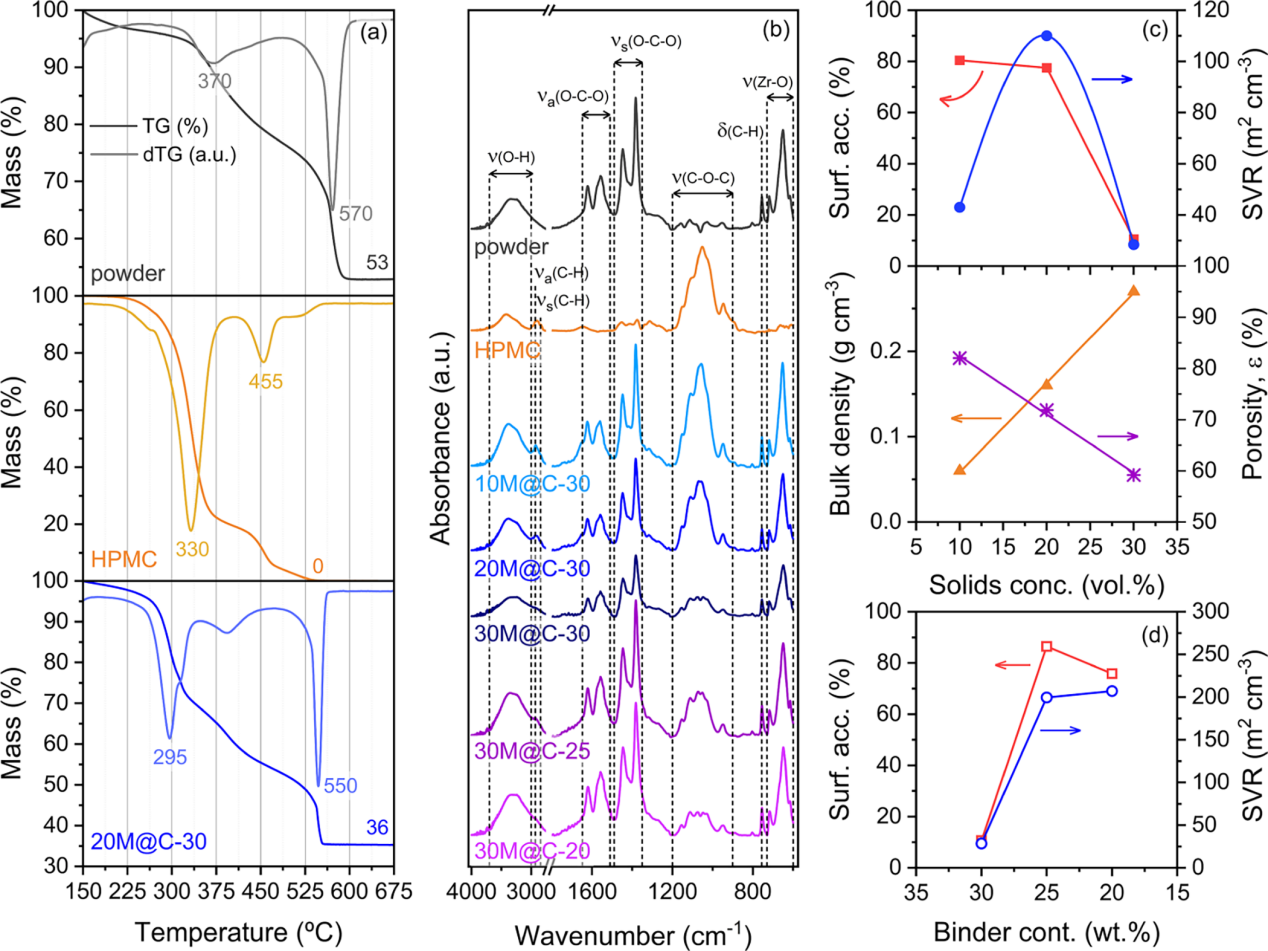
Characterization of MOF-808 powder, hydroxypropyl methylcellulose (HPMC) binder, and MOF-808 beads: (a) TG/dTG profiles, (b) FTIR spectra, and evolution of textural parameters (surface accessibility, surface-to-volume ratio (*SVR*) and porosity (ε)) as a function of (c) solids concentration and (d) binder content.

By contrast, the complete decomposition of HPMC into volatile compounds takes place between 250 and 500 °C, exhibiting dTG peaks at 330 and 455 °C (Figure 2a). Although the two-step thermal decomposition indicates that the employed commercial methylcellulose is somewhat heterogeneous,^39^ it should be noted that the onset decomposition is quite high and within the range of those reported in literature.^39–41^

Notably, TG profiles of *x*M@C-30 (*x* = 10, 20, or 30 vol %) beads can be considered a weight-average of those of MOF-808 powder and HPMC binder, showing several mass losses from 150 to 550 °C that correlate with those observed for the individual components (Figure 2a). It should be noted that a slight shift to lower temperatures is observed for the dTG peaks attributed to BTC groups (570 vs 550 °C) and HPMC decomposition (330 vs 295 °C) of 20M@C-30 beads. This shift may be related to an enhancement in the diffusion of oxidizing gas (air) within the open/porous structure, which alters the decomposition kinetics (leading to lower *T*_max_ and narrower dTG peaks). Likewise, the better dispersion of HPMC within the porous beads (as thin coatings or internal films) makes it more accessible to air, favoring earlier decomposition compared to the bulk binder sample. In addition, interactions between HPMC and the Zr-based MOF surface may also catalyze polymer decomposition and thus lower the observed decomposition temperature. In this line, beads prepared from suspensions with increasing solids loading (Figure S3b–d) show a progressive decrease and broadening of the dTG peaks related to HPMC, which is in agreement with reduced porosity and slower diffusion of air. Overall, taking into account an onset decomposition temperature of ≈250 °C for HPMC, the beads can be considered thermally stable up to 200 °C. Finally, the binder contents (*X*_HPMC_), estimated on a dry basis from TG mass residuals (eq 1), are summarized in Table 2.

**Table 2:** Binder Content and Textural Properties of MOF-808 Powder and Beads with Varying Solid Fractions and Binder Content

Sample	*X*_HPMC_ ^a^ (wt %)	*SSA* ^b^ (m^2^ g^–1^)	*V*_pore_ ^c^ (cm^3^ g^–1^)	*NSSA* ^d^ (m^2^ g_MOF_^–1^)	*NV*_pore_ ^d^(cm^3^ g_MOF_^–1^)	Surf. acc.^e^ (%)	*SVR* ^f^ (m^2^ cm^–3^)
powder	n.a.	1315.8	0.643	1316	0.643	n.a.	809
10M@C-30	30	740.9	0.348	1058	0.497	80	43
20M@C-30	33	683.1	0.315	1020	0.470	77	110
30M@C-30	25	104.5	0.058	139	0.077	11	28
30M@C-25	21	898.7	0.432	1138	0.547	86	200
30M@C-20	17	828.3	0.396	998	0.477	76	207

a Estimated from TG residuals using eq 1.

b Calculated by applying the BET equation.

c Determined from N_2_ uptake at *P*/*P*_0_ ≈ 0.97.

d Surface area or pore volume normalized to the mass of MOF.

e Accessible MOF surface area (%) estimated by eq 2.

f Surface-to-volume ratio calculated by multiplying SSA and bulk density (Table 1).

With the aim of characterizing the functional groups and determining possible chemical interactions between MOF-808 and the HPMC binder, infrared spectroscopy was carried out. FTIR spectra of MOF-808 powder, HPMC binder, and MOF-808 beads are displayed in Figure 2b. The spectrum of MOF-808 shows a broad band from 3700 to 3000 cm^–1^, which is associated with the presence of adsorbed water or structural water interacting with OH^–^ groups of the organic ligand.^36,42^ Besides, coupled bands at 1621/1557 cm^–1^ and 1448/1383 cm^–1^ can clearly be observed, corresponding to asymmetric and symmetric O–C–O stretching vibration modes of carboxyl groups of BTC^3–^ ligands coordinated to Zr^2+^ oxoclusters, respectively.^42–44^ Noteworthy, the absence of a band at around 1700 cm^–1^, attributed to ν­(C=O) vibration mode of free carboxylate, suggests that there is no residual free BTC^3–^ in the sample.^43,45^ While the band at 755 cm^–1^ is most probably associated with out-of-plane bending vibration of C–H groups,^42^ the last two observed bands are related to Zr–O bond stretching vibrations of Zr_6_ nodes.^36,42–44^ In contrast, the spectrum of the HPMC binder shows bands located in other positions and regions. The small bands at 2930 and 2840 cm^–1^, not shown for MOF-808 powder, are due to asymmetric and symmetric H–C–H stretching vibration modes of CH_2_ groups of HPMC.^46^ Instead, the intense band at 1050 cm^–1^ with shoulders at 1100 and 1150 cm^–1^ corresponds to out-of-phase C–O–C stretching vibrations from the glycosidic linkages and hydroxypropyl or methyl groups, respectively.^47–49^ Likewise, the small band at 943 cm^–1^ may be associated with in-phase C–O–C vibrations of ether linkages.^49^ As shown in Figure 2b, the FTIR spectrum of the MOF-808 beads can be considered the result of the superposition of the MOF-808 and HPMC spectra. In fact, all bands appear in almost the same positions, regardless of the solids fraction. Thus, the integration of both components does not affect their IR-vibrational modes. It is noteworthy that if any interaction between both components is occurring, this is likely to happen at the surface of the MOF and polymer particles/scaffold, which could have a small contribution to the overall IR spectrum. However, note that the presence of adsorbed water (band at 3700–3000 cm^–1^) hides the bands corresponding to OH^–1^ groups vibrations, not allowing the identification of possible wavenumber shifts of the OH^–^ stretching vibration bands as a result of hydrogen bonding.^16,50,51^ In fact, this band is difficult to resolve even in the MOF-808 powdered sample after its activation at different temperatures (Figure S5). The presence of hydrogen-bonded H_2_O/OH pairs in the Zr_6_ clusters of MOF-808 can explain the broadening of the IR signal in this region, even when the sample is desolvated at high temperatures.

Once the thermal stability and functional groups of the MOF-808 powder and beads were evaluated, their textural properties (specific surface area (*SSA*) and pore volume (*V*_pore_)) were determined by N_2_ physisorption. Figures 4a and S6 display N_2_ adsorption–desorption isotherms, while textural properties are summarized in Table 2. In addition, Figure 2c exhibits the variation of surface accessibility values and surface-to-volume ratios (*SVR*) as a function of solids loading. From the shape of the isotherms (type I according to IUPAC),^32,52^ it can be deduced that MOF-808 powder is a microporous solid with a two-step micropore filling at relative pressures below 0.1, related to the two types of pores found in its structure (i.e., 4.9 and 18.8 Å). Above this pressure, adsorption barely occurs, with a plateau observed at around 400 cm^3^/g. In this line, the absence of a hysteresis loop at relative pressures above 0.6 indicates that it does not contain mesopores.^43,53^ In fact, the pore size distribution curve of MOF-808 (Figure S7) displays no peaks between 2 and 50 nm (mesopore region according to IUPAC classification) but instead exhibits a sharp one at around 1.2 nm, which lies outside the reliable detection limit, confirming that pore volume originates mainly from micropores. In this line, its *SSA* and *V*_pore_ are quite high (1316 m^2^ g^–1^ and 0.643 cm^3^ g^–1^, respectively) and similar to those reported in the literature.^33,43,52,54^

As a result of combining MOF-808 and HPMC to shape beads with different solid fractions, the N_2_ adsorption capacity clearly diminishes (Figure S6a). This expected decrease is not only due to the introduction of the nonporous binder component (up to 30 wt %), but may also be associated with the complete or partial blockage of the MOF particles by the organic binder (e.g., polyalcohol- and carbohydrate-based binders).^8,25,55–57^ In fact, the textural properties of beads normalized per mass of MOF-808 (*NSSA*) are lower than those of the starting powder (Table 2), evidencing that HPMC reduces the accessibility of N_2_ into MOF, as observed by S. Ohsaki et al.^8^ Despite using the same binder/MOF mass ratio, it was found that the higher the solids concentration of the initial suspension when shaping the beads, the lower the normalized textural properties and surface accessibility (Figure 2c). While 20M@C-30 beads presented slightly lower surface accessibility compared to the 10M@C-30 homologue (77 and 80%, respectively), this was remarkably low for beads prepared from suspensions with 30 vol % solids loading. In this case, *NSSA* and *NV*_pore_ decreased from 1316 to 139 m^2^/g_MOF_ and from 0.643 to 0.077 cm^3^/g_MOF_, indicating that only around 11% of the MOF-808 surface remained accessible. Likewise, this sample also presents the lowest surface-to-volume ratio (Figure 2c) despite containing the highest solids fraction. In agreement with XRD results, the drastic *NSSA* decrease observed for the 30M@C-30 sample could be directly associated with excessive binder coating, resulting from increased particle agglomeration. Note that coating is expected to intensify with increasing solids concentration of the suspension, leading to severe pore blockage.

Afterward, the shape uniformity, porous structure, and macropore size distribution of MOF-808 beads prepared by freeze spherification were evaluated by optical microscopy (OM) and scanning electron microscopy (SEM). Figure 3a–c displays the OM images of MOF-808 shaped by freeze spherification. Regardless of the solids fraction, the beads presented a homogeneous shape with an average diameter of 2.7 mm. While beads with 10 and 20 vol % exhibited a spherical shape, the shape of 30M@C-30 beads was slightly oval due to the deformation of droplets during the dropping. Note that 20 vol % beads presented some little defects (cracks and craters), which might have formed during freeze-drying due to inhomogeneities in the MOF-808/water/HPMC suspension. As expected, the bulk or packing density of the beads increased with the solid fraction, ranging from 0.06 to 0.27 g cm^–3^ (Figure 2c).

On the other hand, SEM images of MOF-808 powder, along with both surface and cross-section views of MOF-808 beads with different solid fractions, are displayed in Figures S8 and Figure 3d–f, respectively. In addition, Table 3 summarizes the results obtained from mercury porosimetry. As shown in Figure S8a, the MOF-808 powder is formed by particles with an octahedral morphology and an average diameter of 1 ± 0.29 μm, which present some extent of agglomeration. On the other hand, it can be visualized that the beads present similar porous structures formed by MOF particles entrapped in HPMC-welted polymeric walls, which are organized to form an interconnected 3D porous structure (see Figure 3d–f). In general, the surface is much less porous than the inner cross-sections of the beads, regardless of the solids loading of the suspension. This may be attributed to the anisotropic radial growth pattern of the ice crystal front during freeze casting: very fast upon initial surface contact with liquid nitrogen, forming small ice nucleation sites,^11,23^ and slowing down as it approaches the nucleus, resulting in a broader ice front. Thus, in the case of 10M@C-30 beads (Figure S8b), the surface contains small and randomly distributed pores of around 1.6 μm, whereas the cross-section contains perfectly oriented lamellar macropores of 12.4 μm, which are formed as a result of ice crystal sublimation during freeze-drying.^19,22^ Since macropore size is inversely related to the freezing rate,^19,20^ the observed pores fall within the small macropore range reported in the literature,^11^ as liquid N_2_ is employed as the freezing agent. Besides, note that well-dispersed MOF particles seem to be adhered onto equally spaced HPMC walls, presumably by physical entanglement and van der Waals interactions,^16^ in agreement with FTIR results. Moreover, it is important to highlight that the macropore size distribution determined by mercury porosimetry aligns well with the pore size estimated by SEM, showing a wide and multimodal size distribution (Figure 3g) as a result of a low solid fraction and interconnected dendrite-shaped pore formation. Mercury intrusion measurements yielded a porosity (ϵ) of 82% (corresponding to a solids fraction of 18%) and an average macropore size of 4.7 μm.

**Table 3:** Porosity, Macropore Volume, and Macropore Size of MOF-808 Beads Determined by Mercury Porosimetry

Sample	Porosity, ε^a^ (%)	*V*_macro_ ^a^ (cm^3^ g^–1^)	Mdn. pore size^b^ (μm)	Avg. pore size^c^ (μm)
10M@C-30	82	12.74	31.2	4.7
20M@C-30	72	3.35	8.7	2.8
30M@C-30	59	2.63	28.9	14.0
30M@C-25	67	2.07	2.2	1.7

a Porosity (excluding micropores) and macropore volume determined from total Hg intrusion.

b Median macropore diameter (volume) at 5.79 psia and 6.37 mL/g.

c Average macropore diameter (4 V/A).

20M@C-30 bead surface also presents random and low porosity (average SEM pore size of 4.3 μm). It is worthy to mention that a small part of the inner porous structure of this bead appears to be cellular rather than lamellar, suggesting localized random or isotropic growth of ice crystals (Figure 3e). Interestingly, similar porous structures have been visualized for MOF@cellulose aerogels or foams prepared by sol–gel combined with freeze-drying.^51,58,59^ Although some agglomerates are observed as a consequence of higher solids loading of the suspension, MOF seems to be well distributed throughout the HPMC matrix. In this line, the macropore size distribution was observed to be multimodal but narrower (Figure 3h) than that of 10M@C-30 beads and shifted toward smaller pore sizes due to the two-times higher solids fraction (Table 1), leading to lower porosity and average macropore size (Table 3).

Upon further increasing the solids loading of the suspension to 30 vol %, the surface porosity is dramatically reduced (see Figure S8d). In fact, the 30M@C-30 bead seems to be covered by a commercial methylcellulose shell, making it barely possible to observe pores by SEM (i.e., the presence of mesopores cannot be ruled out). The lack of pores on the surface of the 30M@C-30 beads, together with a high encapsulation degree of the MOF particles by the polymer, would explain the drastic reduction of the accessible surface area of this sample (Figure 2c). In this context, it is important to highlight that the significantly lower surface porosity compared to the inner porosity in all cases may not only result from the dynamic growth of the ice front but also from the partial exudation of HPMC during the initial freezing stage, leading to partial blockage of surface pores in the beads. Unexpectedly, the porous structure of the inner cross-section appears to be equiaxial,^11^ consisting of homogeneous and larger circular pores of around 14 μm, which is consistent with the average size determined by mercury intrusion porosimetry (Table 3). Besides, the pore size distribution is notably narrower, probably due to the highest solids fraction, and exhibits an unimodal profile centered at around 8 μm (Figure 3i). The presence of bigger pores in the case of beads with the highest solids fraction is explained by the formation of significantly wider (>2 μm) walls. Note that these walls are formed by highly HPMC-coated or embedded MOF particle agglomerates, which is also in agreement with the poor surface accessibility observed for these beads and suggests that the binder content (30 wt %) is excessive to shape beads from suspensions with 30 vol % solids loading.

### 3.2 Influence of Binder Content

Achieving an optimal balance between bulk density (or solids fraction) and macroporosity of beads is crucial to ensure effective internal diffusion (or surface accessibility) while minimizing bed volume. In this sense, beads with higher bulk density or solids fraction seem to be more suitable for adsorption or catalysis purposes, despite presenting poorer textural properties. Thus, in an attempt to increase the *SVR* of beads prepared from suspensions with 30 vol % solids concentration (Table 2), 30M@C-25 and 30M@C-20 samples were synthesized by lowering the binder content to 25 and 20 wt %, while maintaining the solids concentration of the suspension. The shape of the new MOF-808 beads was spherical and quite homogeneous, with average sizes of around 2.8 and 2.9 mm.

As in the previous cases, the crystallinity of MOF-808 was preserved after the shaping process, although slightly broader peaks (higher FWHM) were observed compared to those of the starting powder (Figure S1). Noteworthy, beads with the intermediate HPMC loading (25 wt %) exhibited the lowest signal of the polymeric component in the PXRD patterns, as shown in Figure 1b. In addition, TG profiles of 30M@C-25 and 30M@C-20 samples proved to be similar to those of 30M@C-30 beads (Figure S3d–f). It should be noted that no clear relation between binder content and peak positions could be established, since decomposition temperature may also be influenced by other factors such as binder distribution, pore connectivity, and interactions. The binder contents, calculated by eq 1, were 21 and 17 wt %. Furthermore, the FTIR spectra were comparable to those of beads with different solids fractions (Figure 2b), with no new bands or shifts indicating chemical bonding between MOF-808 and HPMC.

The textural properties, along with MOF accessibility and surface-to-volume ratios obtained from N_2_ adsorption isotherms, are summarized in Table 2. Noteworthy, by decreasing the binder content from 30 to 25 wt %, the *NSSA* significantly increased from 139 to 1138 m^2^/g_MOF_^–1^. This suggests that, unlike the 30M@C-30 beads, the binder concentration is not high enough to coat much of the MOF particles of 30M@C-25 beads, resulting in a remarkable MOF surface accessibility of 86% and ≈ 7 times higher *SVR* ratio (200 m^2^/cm^3^, as shown in Figure 2d). This confirms that a 30 wt % binder content was excessive for the shaping of beads prepared from suspensions with 30 vol % solids loading.

However, despite containing less binder, 30M@C-20 beads present slightly lower surface accessibility (76%). This may be attributed to additional pore blockage of MOF-808 during the mechanical densification process of 30M@C-20 beads. Furthermore, the mechanical integrity of the beads was qualitatively assessed to evaluate the effect of the binder content. The 30M@C-20 beads exhibited poor structural stability, fracturing under compression with tweezers during routine handling. This suggests that a 20 wt % binder content is insufficient to establish a mechanically robust MOF-808@HPMC macroporous network, a finding consistent with studies on other porous composites where a critical binder threshold is required for structural stability.^60^ In contrast, the 30M@C-25 beads exhibited sufficient mechanical robustness, withstanding manipulation without fracturing. This enhanced integrity is a critical attribute for their potential deployment in packed-bed applications, where mechanical strength is necessary to prevent attrition and pressure drop issues.^61,62^ Therefore, it can be concluded that 25 wt % is the optimum binder content among those studied to shape MOF-808 beads prepared from suspensions with 30 vol % solids loading.

Noteworthy, in terms of specific surface area (*SSA*) preservation, freeze spherification performs comparably with conventional methods. In the case of pelletization, *SSA* and pore volume losses are highly dependent on the applied pressure and the mechanical robustness of the MOF framework. For robust MOFs (e.g., Zr-based frameworks), mechanically stable pellets can be obtained using compression pressures around 100–150 MPa, typically resulting in SSA losses of no more than 10–15%.^6,7,56,63^ In contrast, more fragile MOFs (e.g., MIL-53) may exhibit SSA losses of up to 43% under the same conditions.^6^ In the case of granulation, as with freeze spherification, the SSA loss is influenced by binder content and type.^8,9,55,64^ Higher binder content reduces the overall MOF fraction in the shaped material, thereby decreasing textural properties.^8^ Furthermore, certain binders may coat the MOF particles more extensively, further reducing surface accessibility. In our case, the optimal formulation achieved SSA preservation above 85%, which is in line with or better than values reported for other granulation approaches using moderate binder contents.^9,64^

Additionally, as a second accessibility measurement, CO_2_ adsorption experiments were carried out for the MOF-808 powder and 30M@C-25 beads. The isotherms, measured at 273 K, are displayed in Figure 4b. In general, it can be observed that the amount of adsorbed CO_2_ increases with equilibrium pressure, following a trend toward saturation but without reaching full saturation. The CO_2_ uptake of MOF-808 powder at an equilibrium pressure of 1 bar resulted in 1.86 mmol g^–1^ (81.8 mg CO_2_ g^–1^), which is reasonably higher and in agreement with CO_2_ adsorption uptakes reported for MOF-808 samples measured at higher temperatures of 298 K (1.35 mmol g^–1^)^65^ and 328 K (1.00 mmol g^–1^).^66^ Moreover, the isotherm was fitted to both Langmuir and Freundlich models, obtaining a better fit or a higher coefficient of correlation (*R* ^2^ for the Freundlich model (0.964 vs 0.996), which indicates that non ideal or heterogeneous CO_2_ adsorption takes place on MOF-808 as a result of sites with different affinities for CO_2_ (*n* = 1.2).^67^ It should be highlighted that the shapes of the isotherms for 30M@C-25 beads are similar, achieving a CO_2_ uptake of 1.54 mmol g^–1^ or 2.01 mmol g_MOF_^–1^ at 1 bar equilibrium pressure. The fact that the normalized CO_2_ uptake is slightly higher than that of the powder (1.86 mmol g^–1^) confirms that entrapped MOF-808 is fully accessible and may also suggest that the HPMC binder is also able to adsorb CO_2_.

The porous structure of 30M@C-25 beads was also assessed by SEM. Surface and cross-sectional views of 30M@C-25 beads are included in Figures S9c and 4c, respectively. In general, a much more open porous structure with more accessible MOF particles is visualized for 30M@C-25 beads in comparison to that of 30M@C-30 beads (see Figure 3f). The textural information obtained from SEM agrees with the higher accessibility to the MOF-808 particles observed by N_2_ and CO_2_ adsorption. In fact, the surface of the beads presents a notably higher number of randomly distributed pores with a size of 1.97 μm (Figure S9c), whereas the cross-section contains lamellar-oriented pores of 4.31 μm (Figure 4c). Likewise, the macropore size distribution is narrow and unimodal (Figure 4d), obtaining an average macropore size and porosity of 1.7 μm and 66%. Note that the pores have different morphologies and are smaller than those of 30M@C-30 beads, which indicates that dendrites could form properly when employing less binder.

**Figure 3:**
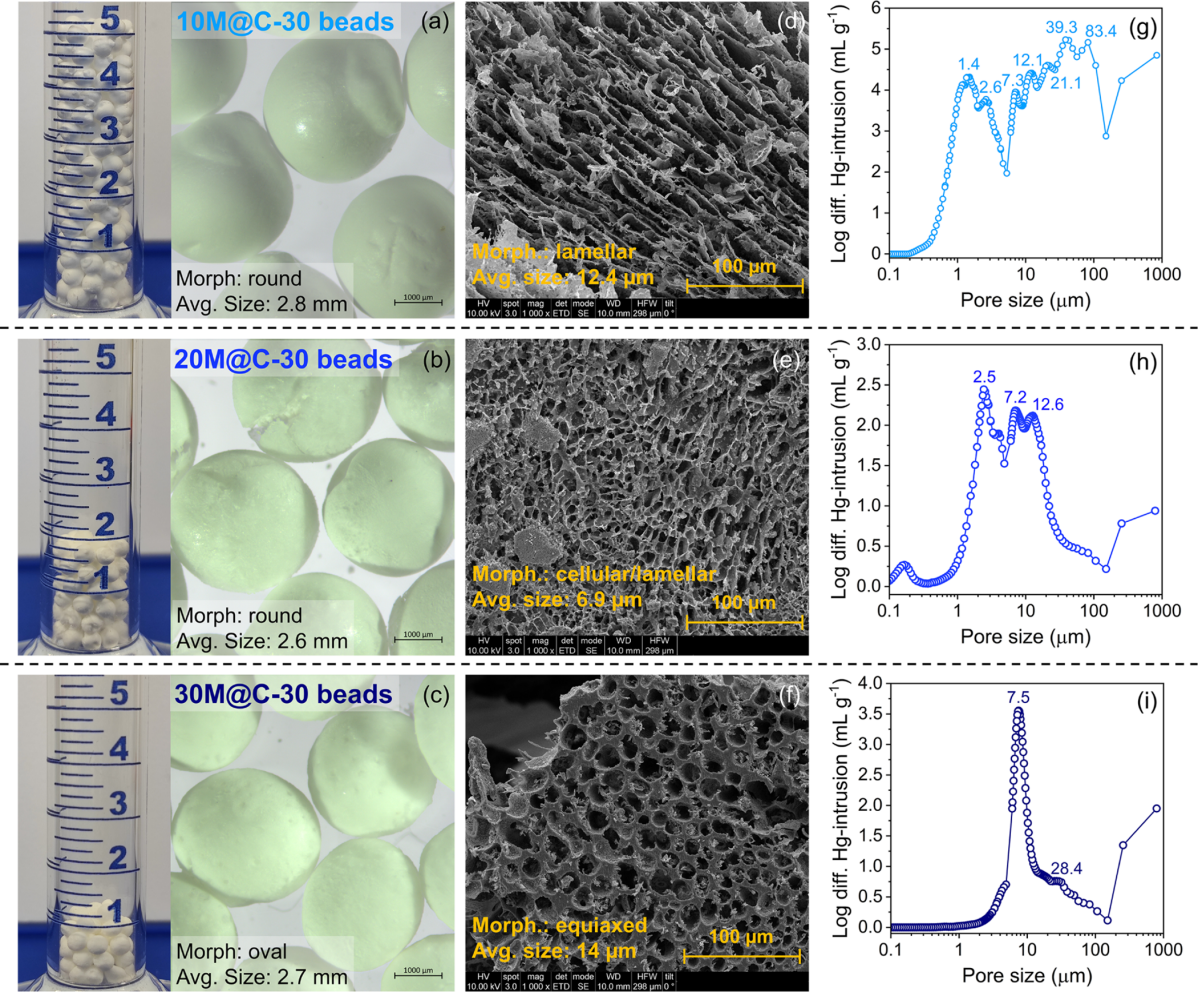
(a–c) Optical images, (d–f) SEM micrographs of cross-section views, and (g–i) macropore size distribution histograms of MOF-808 beads prepared from suspensions with increasing solids loading (10, 20, or 30 vol %).

**Figure 4:**
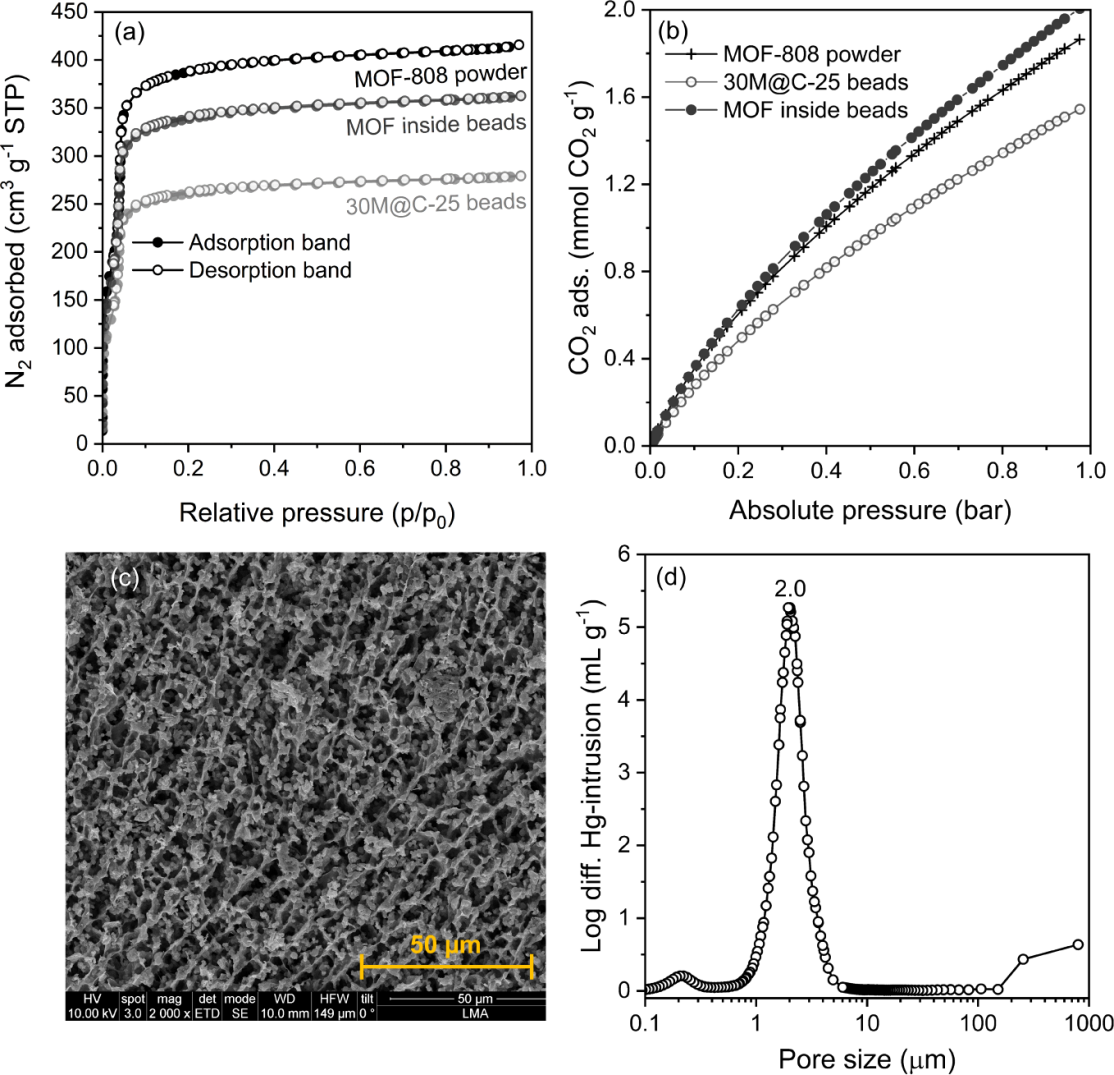
Characterization of 30M@C-25 beads: (a) N_2_ adsorption–desorption isotherms recorded at 77 K, (b) CO_2_ adsorption isotherms recorded at 273 K, (c) SEM micrograph of cross-section view, and (d) macropore size distribution histogram determined by mercury intrusion porosimetry.

Finally, to determine the versatility of this method, UiO-66-NH_2_ and MIL-100­(Fe) MOFs were shaped by freeze spherification into beads, employing the best formulation (suspension solids loading of 30 vol % and 25 wt % binder). The results are included in the Figures S10–S13. As in the case of MOF-808, the crystallinity of UiO-66-NH_2_ and MIL-100­(Fe) was not significantly affected by the shaping process (Figure S10) and according to TGA results, the beads are thermally stable up to 150 and 200 °C, respectively (Figure S11). Regarding the textural properties, no remarkable specific surface area decrease was observed, with slightly higher accessibilities of 88 and 86%, respectively. Both samples also presented an open and oriented lamellar macroporous structure similar to that of 30M@C-25 beads (Figure S13), evidencing that this method can be used for other MOFs.

## 4 Conclusions

In this work, the MOF powder was successfully shaped into beads by freeze spherification, an easily scalable and novel method derived from freeze casting. Unlike classic shaping techniques, this approach allows the formation of beads with hierarchical porosity, enhancing diffusion properties. Among other things, we particularly shaped MOF-808 due to its high thermal stability and ease of functionalization, varying the solids loading and binder content of the initial MOF/HPMC aqueous suspension. The method allowed producing uniform spherical MOF-808 beads at lower suspension solid loadings and transitioning to oval shapes as the solids content increases.

To study the effect of solids loading in the suspension, the binder content was fixed at 30 wt %. In any case, the combination of MOF-808 with HPMC during the shaping process did not significantly affect the crystallinity, thermal stability, or functional groups of MOF-808, concluding that MOF particle adhesion into the HPMC matrix is physical rather than chemical. However, increasing the solids concentration of the suspension, specifically from 20 to 30 vol %, led to a significant reduction in the MOF’s accessible surface area (from 77 to 11%) due to a drastic combination of solids agglomeration and the binder coating effect. On the other hand, the porous structure of 10M@C-30 and 20M@C-30 beads consisted of oriented lamellar morphology as a result of ice dendrite sublimation, with 20M@C-30 beads exhibiting smaller pore sizes and narrower macropore distribution due to two times higher solids fraction. However, 30M@C-30 beads showed a different porous structure, characterized by fewer tubular macropores and thicker MOF particle agglomerates, evidencing that the binder concentration for this sample was excessive.

Next, the binder content was reduced for 30 vol % beads to investigate its effect on surface area and structural stability. A decrease in binder content from 30 to 25 wt % resulted in a remarkable increase of accessible surface area (from 11 to 86%) due to reduced HPMC binder coating. However, beads formulated with 20 wt % of binder (30M@C-20) exhibited poor structural stability and were not robust enough for handling and manipulation, indicating an insufficient quantity of HPMC to effectively bind the MOF particles into a cohesive scaffold. Therefore, suspension solids loading of 30 vol % and binder content of 25 wt % proved to be optimal freeze spherification conditions among those studied, enabling the formation of beads with high surface accessibility (86%), surface-to-volume ratio (200 m^2^ cm^–3^), sufficient mechanical integrity, and a well-interconnected porous structure.

Overall, this study also demonstrated the potential and applicability of freeze spherification in shaping other powdery MOFs (UiO-66-NH_2_ and MIL-100­(Fe)) to generate hierarchical porosity bead structures, offering insights into the optimization of bead composition and the effects of shaping parameters.

## Supplementary Material



## Data Availability

All relevant data are presented in the main text and in the electronic Supporting Information (ESI).

## Notes

Contains detailed experimental procedures for the synthesis and shaping of the Metal–Organic Framework (MOF) materials. It also provides supplementary physicochemical characterization data for all prepared powders and shaped beads. This data includes powder X-ray diffraction (PXRD) patterns and corresponding Rietveld analysis plots, thermogravimetric (TG) and derivative thermogravimetric (dTG) profiles.^1^ H NMR and Fourier-transform infrared (FTIR) spectra. N_2_ physisorption isotherms, pore size distributions, and textural properties. Scanning electron microscopy (SEM) micrographs (PDF)
