# Supplementary material for: Shaping Hierarchical Porosity Beads from Metal–Organic Framework Powders Using Freeze Spherification
Source: Chem Mater. 2025 Oct 30;37(21):8501–13. doi: 10.1021/acs.chemmater.5c00901 (PMC12614042; doi:10.1021/acs.chemmater.5c00901)
Supplement: Supplementary file 1 [file cm5c00901_si_001.pdf]

SUPPORTING INFORMATION

Shaping Hierarchical Porosity Beads from Metal-Organic Framework Powders Using Freeze Spherification

Adrián Quindimil[a], Roberto Fernández de Luis[b], Stefan Wuttke[c], Jonas Gurauskis[a, d,*].

[a]*Instituto de Nanociencia y Materiales de Aragón (INMA), Consejo Superior de Investigaciones Científicas (CSIC)–Universidad de Zaragoza (UNIZAR), Calle Mariano Esquillor 15, CIRCE Building, 50018 Zaragoza, Spain.*

[b]*BCMaterials, Basque Center for Materials, Applications and Nanostructures, UPV/EHU Science Park, 48940 Leioa, Spain*

[c]*Academic Centre for Materials and Nanotechnology, AGH University of Krakow, 30-059 Krakow, Poland.*

[d]*Fundación Agencia Aragonesa para la Investigación y el Desarrollo (ARAID), Avenida de Ranillas 1D, 50018 Zaragoza, Spain.*

*jonas.gurauskis@csic.es

Table of Contents

S1. Additional results on MOF-808 powder and beads 1

S1.1. Preparation of MOF-808 beads by freeze spherication 1

S1.2. Characterization results 1

S2. Shaping of UiO-66-NH_2 and MIL-100(Fe) by freeze spherification 10

S2.1. Experimental 10

S2.1.1. Synthesis of gel-based UiO-66-NH2 and MIL-100(Fe) powder 10

S2.1.2. Shaping by freeze spherification 10

S2.1.3. Characterization techniques 10

S2.2. Characterization results 10

S1. Additional results on MOF-808 powder and beads

S1.1. Preparation of MOF-808 beads by freeze spherication

Porous MOF-808 beads with increasing solids volume fraction were prepared by freeze spherification method, varying the total solids (MOF and binder) loading (10, 20 or 30 vol.%) of the initial suspension. Commercial hydroxypropyl methylcellulose (MethoCel K3 Premium LV) from *ChemPoint* was employed as binder, and water as solvent. As shown in **Scheme S1**, the aqueous suspension was prepared at first by dispersing the required amount of MOF-808 powder into HPMC/H_2O solution under vigorous stirring (1000 rpm) for 1 h. Crystallographic densities of 0.902 g cm^{-3} (MOF-808) and 1.330 g cm^{-3} (HPMC) were assumed for solids loading calculations. Afterwards, the suspension (5–10 mL) was added dropwise through a 1.5 mm ID tube connected to a peristaltic pump into a liquid N_2 bath (1 L, 77 K) at a flow rate of 0.15 mL min^{-1}. This flow rate was optimized as the maximum value to prevent drops from sticking to each other. Upon contact with liquid N_2, droplets floated for around 5 s due to Leidenfrost effect before sinking and, once completely frozen, were collected as ice-templated MOF-808@HPMC beads. Finally, the beads were freeze dried in a *Telstar* lyophilizer at -42 °C and 0.5 mbar for 24 h. The process induces the ice dendrite sublimation and the subsequent formation of a 3D interconnected macropore network. MOF-808 beads are named xM@C-y, where x represents the solids loading (10, 20 or 30 vol.%) of the initial suspension, M@C denotes MOF embedded in cellulose matrix and y denotes the nominal binder (HPMC) content (30, 25 or 20 wt.%).

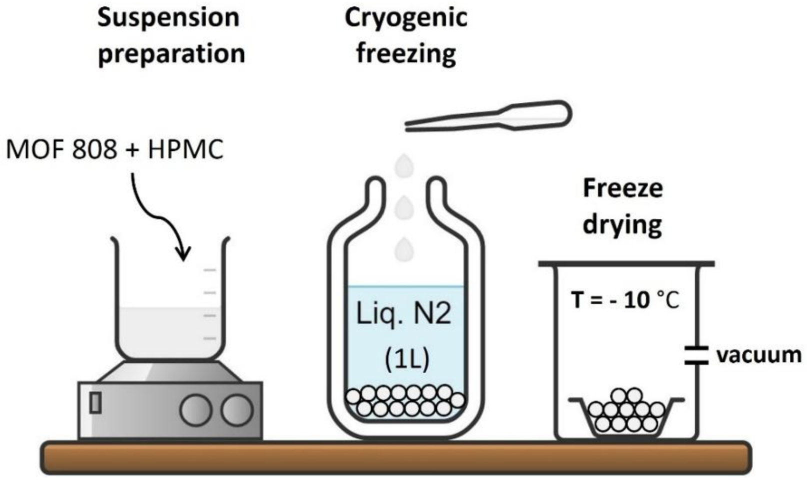

Scheme S1. MOF-808 beads preparation scheme.

S1.2. Characterization results

Figure S1 shows the PXRD patterns of the MOF-808 and HPMC components, as well as the data for the shaped samples, which were previously softly grounded into powder. The simulated pattern from MOF-808 has been included as a reference. The PXRD pattern of the MOF-808 sample presents the characteristic diffraction maxima arising from the long-range ordering of the cubic Fd-3m structure (a= 35.0764 Å) of the MOF-808 material. This structure consists of $Zr_6O_4(\mu_3\text{-}OH)_4(CH_3COO)_6$ clusters coordinated to six BTC^{3-} ligands, which in turn are linked to three Zr clusters, forming a 3D network with tetrahedral (4.8 Å) cages and larger adamantine-shaped (18 Å) cavities.

It is important to note that a preliminary comparison of the experimental and the simulated data for MOF-808 discard the presence of any impurity in the sample. MOF-808 shaped samples show small full width at half maximum values (FWHM), along with small contributions arising from amorphous polymeric components derived from the freeze spherification. Instead, the two broad peaks (i.e. 8.9 and 19.5°in 2θ) of HPMC point that the binder is in general an amorphous solid with a very limited long-range ordering.

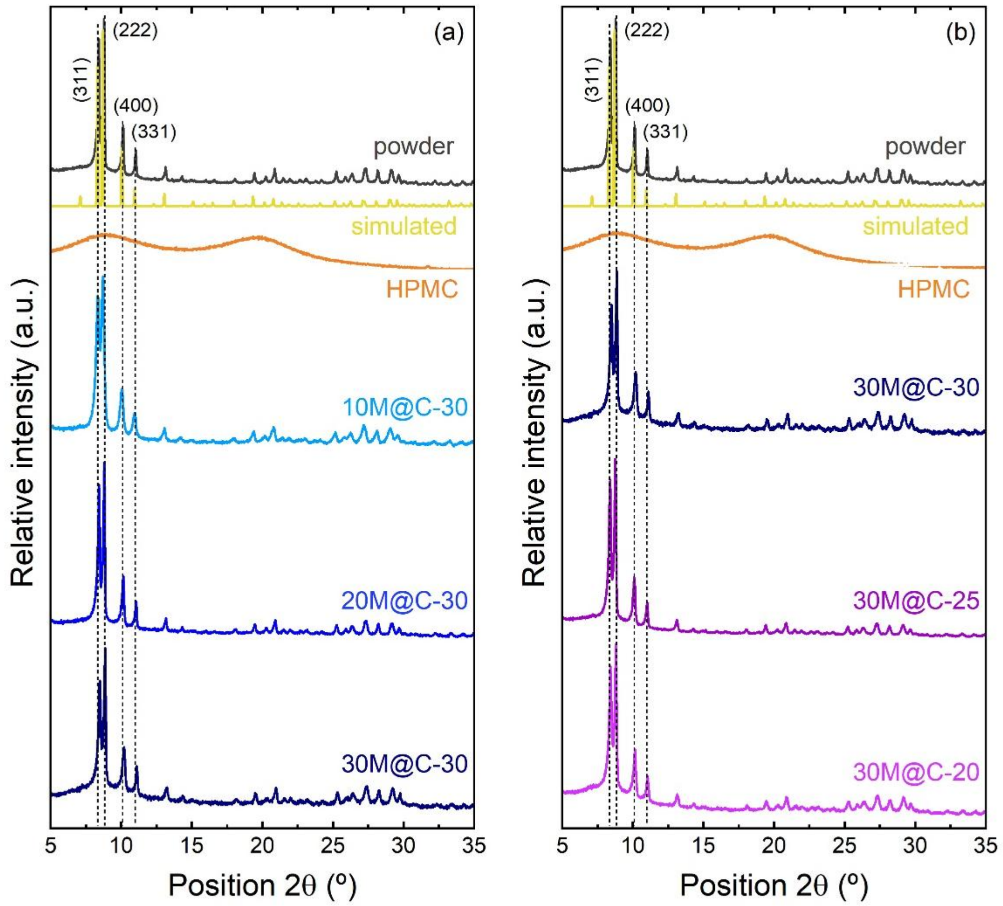

Figure S1. PXRD data for MOF-808, HPMC and freeze-shaped samples prepared from suspensions with (a) increasing solids concentration and (b) with varying binder content. The simulated pattern for MOF-808 has been included as a reference.

In order to analyse the slight variation on the relative intensities of the patterns, the Rietveld refinement of the data was done starting from the structural model of the MOF-808 reported by H. Furukawa et al. The solvent molecules were removed from the structure, and the refinement was done without the modification of the atomic positions of the model. A resolution file – obtained from the refinement of LaB_6 standard – was employed to estimate the average size of the crystalline domains and the strain of the freeze spherificated samples from the Rietveld refinement. As the same batch of MOF-808 was employed to shape all the samples, it is not expected a variation of the size of the crystalline domains. Thus, we employed the value of the size of the crystalline domains obtained for MOF-808 (i.e. 573±2 nm) to the Rietveld refinements of the freeze spherificated samples. In fact, this value agrees with the crystal size shown in scanning electron microscopy images. Thus, any additional broadening of the maxima was ascribed to the increase of the crystallographic strain in the MOF-808 crystals induced by the shaping process.

The final fittings of the PXRD data for the rest of shaped samples after their Rietveld analysis are shown in **Figure S2.** Note that a detail of the fitting for the high-angle region has been included for each fitting.

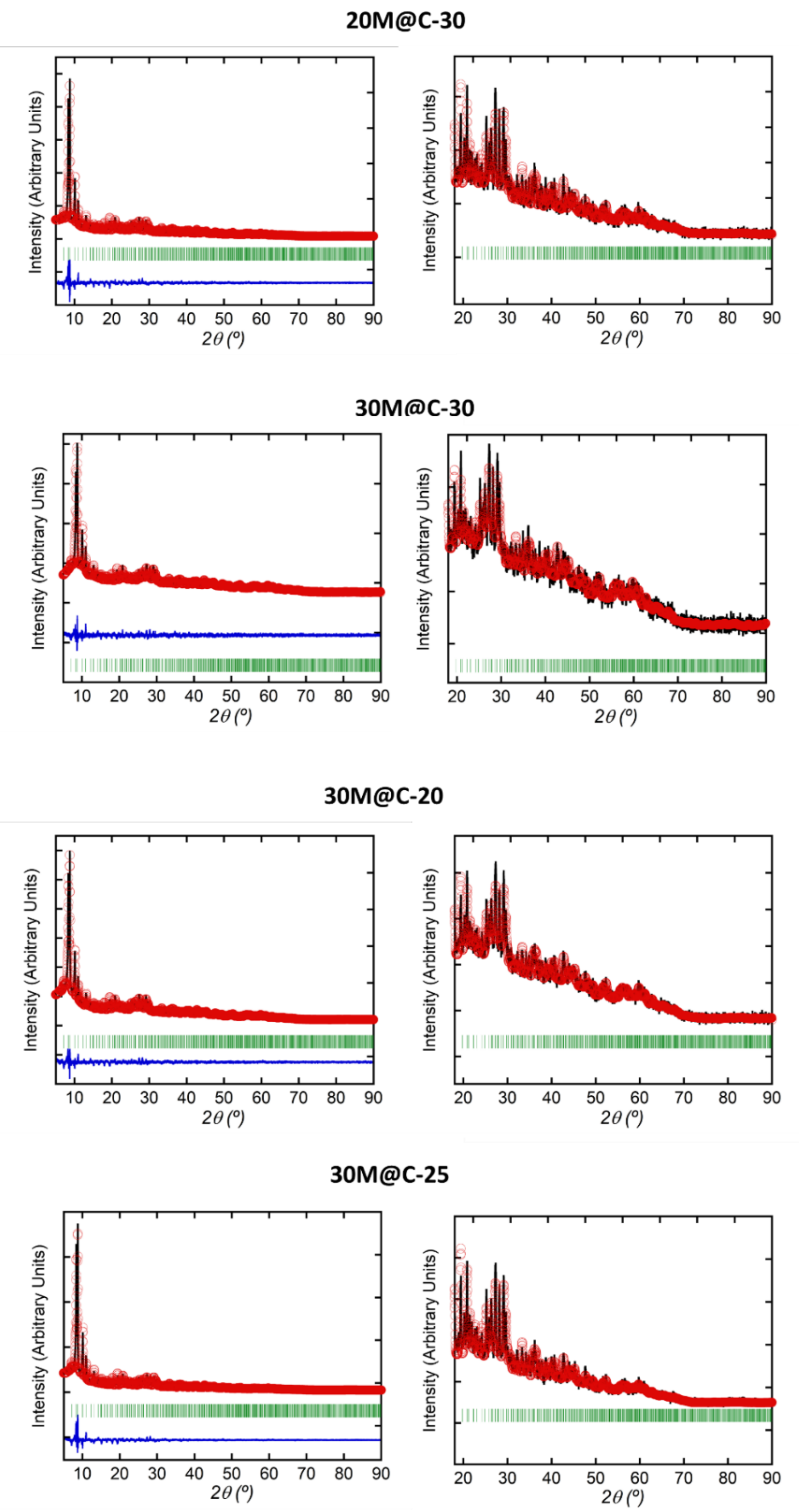

Figure S2. Final fittings of the PXRD data for the shaped samples after their Rietveld analysis. A detail of the fitting for the high-angle region has been included for each fitting. Red circles: experimental data. Black line: calculated pattern. Blue line: difference between the experimental and calculated data. Green Bars: Bragg reflections.

Figure S3 shows the mass loss (TG) and corresponding derivative (dTG) profiles of MOF-808 powder, HPMC binder and beads prepared from suspensions with different solids loading and with varying binder contents. In general, the mass loss occurs in different steps that can be identified by dTG curves. In all cases, the mass loss observed below 150 °C is attributed to solvent removal, with the corresponding water contents summarized in **Table S1**.

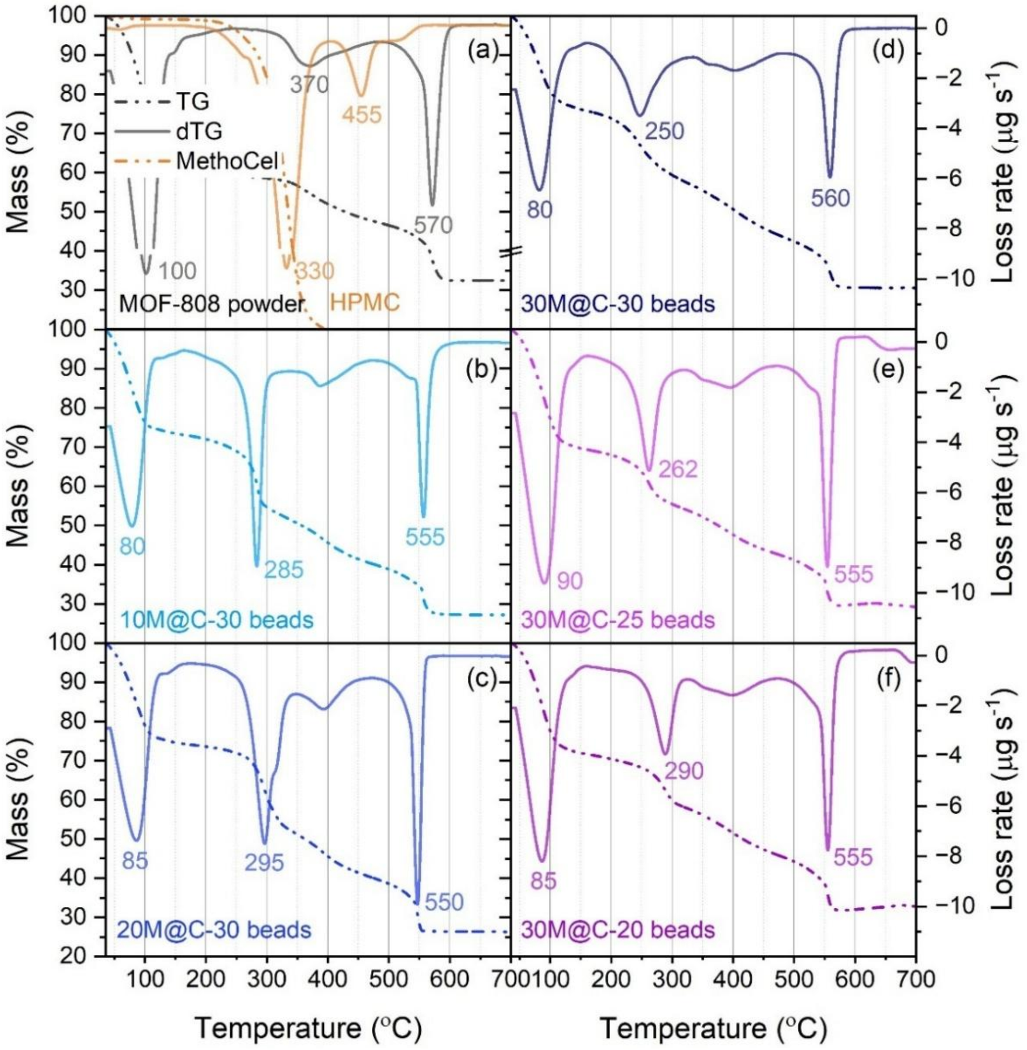

Figure S3. TG and dTG profiles of (a) MOF-808 powder and binder, (b, c, d) beads with increasing solids fraction and (d, e, f) 30 vol.% beads with different binder content.

In the case of MOF-808 powder (**Figure S3a**), the initial mass loss between 150 and 350 °C is associated with release of acetate modulator molecules and the dehydration of the $[Zr_6O_4(OH)_4]^{12+}$ clusters. Above 350 °C, mass loss became more pronounced due to calcination of the trimesate linkers. Note that the dTG peak at around 570 °C may be attributed to combustion of remaining organic compounds, leading to pronounced final mass loss. By contrast, the complete decomposition of HPMC into volatile compounds takes place between 250 and 500 °C, exhibiting dTG peaks at 330 and 455 °C. MOF-808 was further characterized by ^{1}H-NMR after its digestion in NaOH-1M deuterated solution. As expected, proton shifts recorded in the spectra (**Figure S4**) match with the usual ones of trimesic and acetic acid components. The integration of selected signals indicates a molar ratio of 0.5 to 1.0 of the trimesic acid with respect to the acetic. Taking this information into account, the following average formula, $Zr_6(\mu_{3-}O)_4(\mu_{3-}OH)_4$ $(\mu_{1-}$

$OH)_2(\mu_1\text{-}H_2O)_2(C_9H_3O_6)_2(C_2H_3O_2)_4$, for MOF-808 can be proposed, if the coordination positions in the clusters not occupied by acetate and trimesate molecules are compensated by water/hydroxyl pairs. In fact, considering this formula, a value of the residual weight of 52.6 % after the calcination of the initial material can be calculated, a value that is very close to the experimental one observed on the TGA experiment.

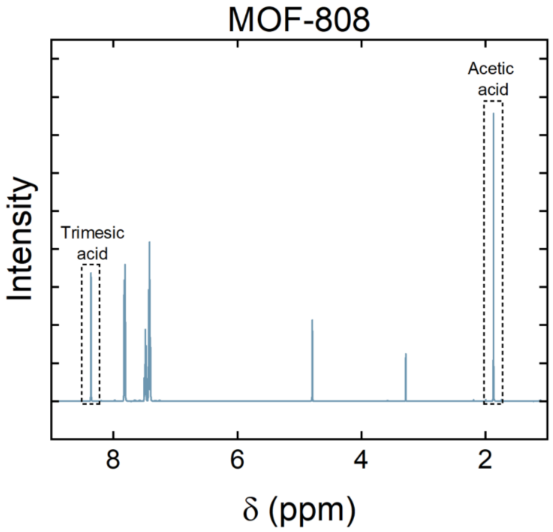

Figure S4. 1-HMR spectra of digested MOF-808 sample in NaOH-1M deuterated water solution.

By contrast, TG and dTG profiles of MOF-808 beads can be considered a weight average of those of MOF-808 powder and HPMC binder, presenting several mass losses from 150 to 550 °C that can be correlated to the ones observed in individual compounds. It should be noted that, in general, a slight shift to lower temperatures is observed for the dTG peaks attributed to BTC groups (570 vs. 550 °C) and HPMC decomposition (330 vs. 295 °C) of beads due to an enhancement in the diffusion of oxidizing gas (air) within the open/porous structure (leading to lower Tmax and a narrower dTG peak. In this line, for beads prepared from suspensions with increasing solids concentration (**Figures S3b-d**), the intensity of dTG peak of HPMC clearly decreases while simultaneously broadening as the solids fraction increases or porosity decreases. However, no clear relation between binder and peaks position could be stablished (**Figures S3d-f**), since decomposition temperature not only may be influenced by binder content but also by other factors such as binder distribution, pore connectivity and interactions. The binder contents of all beads, estimated on a dry basis by Equation 1, are summarized in the following table:

Table S1. Water and binder (HPMC) content of MOF-808 estimated by TGA.

Sample	X_{H2O}[a] (wt.%)	Residue[b] (wt.%)	X_{HPMC}[c] (wt.%)
powder	39	53	n.a.
10M@C-30	27	37	30
20M@C-30	25	36	33
30M@C-30	24	40	27
30M@C-25	30	42	21
30M@C-20	28	44	17

[a]Mass loss up to 150 °C.
[b]TG residual at 700 °C, calculated on a dry basis (T > 150 °C).
[c]Estimated by Equation 1.

Figure S5 exhibits FTIR specra of MOF-808 sample at different activation temperatures. Note that the presence of adsorbed water (band at 3700-300 cm^{-1}) hides the bands corresponding to OH^{-1} groups vibration, not allowing the identification of possible wavenumber shifts of the OH^{-} stretching vibration bands as a result of hydrogen bonding. In fact, this band it is difficult to resolve even in the MOF-808 powdered sample after its activation at different temperatures (**Figure S5a**). The presence of hydrogen bonded H_2O/OH pairs in the Zr_6 clusters of MOF-808 can explain the broadening of the IR-signal in this region even when the sample is de-solvated at high temperatures.

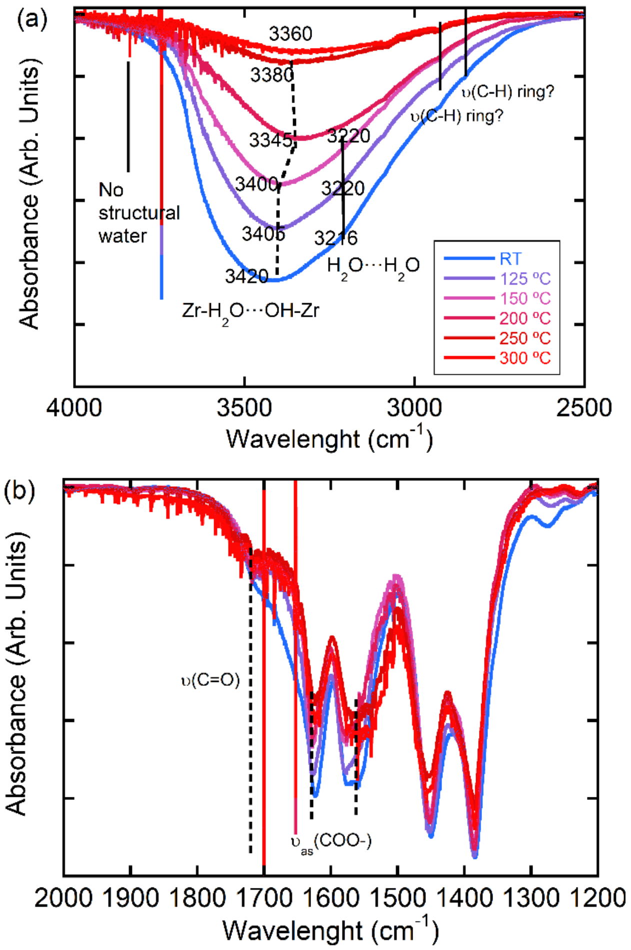

Figure S5. IR spectra of MOF-808 sample obtained after heating the KBr-MOF-808 pellets at different temperatures. The IR-spectra was recorded immediately after the sample was taken from the oven. (a) 4000 to 2500 cm^{-1} region. (b) 2000 to 1200 cm^{-1} region.

N_2 adsorption-desorption isotherms of MOF-808 powder, HPMC binder and beads with different solids loading are displayed in **Figure S6**. The isotherms exhibit a type I shape according to IUPAC clasification, indicating that all samples are microporous solids. On the other hand, the absence of a hysteresis loop at relative pressures above 0.6 indicates that any of them contain mesopores. As a result of combining MOF-808 and HPMC to shape beads with increasing solids loading (10, 20 and 30 vol.%), the N_2 adsorption capacity (in m^2/g) clearly diminishes (**Figure S6a**). This expected decrease is not only due to introduction of non-porous binder component (up to 30 wt.%), but may also be associated with complete or partial blockage of the MOF particles by the organic binder (e.g., polyalcohol and carbohydrate-based binders). In fact, textural properties of beads normalized per mass of MOF-808 are lower than those of starting

powder, evidencing that HPMC reduces the SSA accessibility of N_2 into MOF. Despite having used the same binder/MOF ratio, it is found that the higher the solids loading, the lower the normalized textural properties. The SSA loss was remarkably higher for beads with 30 vol.% solids loading (89%). In this case, SSA and V_{pore} decreased from 1316 to 139 m^2/g and from 0.643 to 0.077cm^3/g, which indicates that only around 11% of MOF-808 surface remained accessible. The drastic SSA decrease observed for 30M@C-30 sample, could be directly associated with an excessive binder coating, resulting from increased particle agglomeration, which is expected to intensify with increasing solids loading of the initial suspension, leading to severe pore blockage.

Figure S6b shows adosorption isotherms of 30 vol. beads with varying binder content. Noteworthy, the N_2 adsorption capacity of 30 vol.% beads clearly grew by only decreasing binder content from 30 to 25 wt.%. Accordingly, the *NSSA* was significantly increased from 139 to 1138 m^2/g_{MOF}^{-1}. This suggests that, unlike for 30M@C-30 beads, the binder concentration is not high enough to coat much of the MOF particles of 30M@C-25 beads, resulting in a remarkable MOF surface accessibility of 86% and ≈ 7 times higher surface-to-volume ratio (200 m^2/cm^3). However, despite containing less binder, 30M@B-20 beads present slightly lower N_2 adsorption capacity (Figure S6b) and surface accessibility (76%). This may be attributed to additional pore blockage of MOF-808 during mechanical densification process of 30M@B-20 beads.

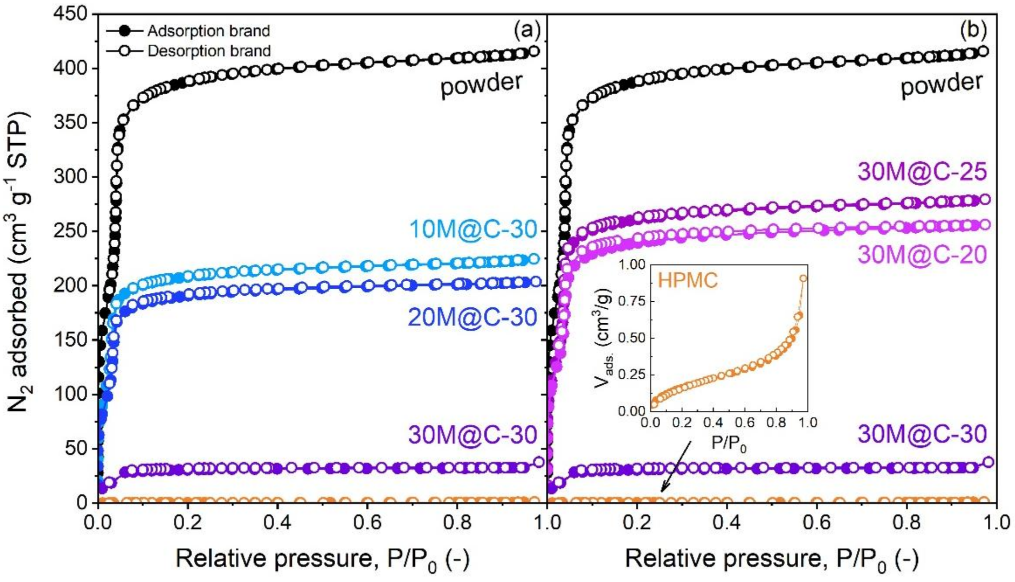

Figure S6. N_2 adsorption-desorption isotherms of MOF-808 powder, HPMC binder and beads with varying (a) solids fraction and (b) binder contents at 77 K.

Figure S7 shows pore size distribution (PSD) curves of MOF-808 beads with increasing solids fraction and binder contents, obtained applying the BJH model. The PSD of MOF-808 powder displays no peaks in the 2-50 nm mesopore region (IUPAC clussification) but instead exhibits a sharp peak at around 1.2 nm, confirming that the pore volume originates mainly from micropores. In the case of beads prepared from suspension with increasing solids loading (**Figure S7a**), pore volume maximum decreases and the peak shifts toward smaller pore sizes, consistent with binder coating effect. Notably, 30M@C-30 samples shows a markedly lower peak intensity, in agreement with its poor N_2 adsorption capacity. The pore volume clearly increases by reducing binder content, being 30M@C-25 sample the one with highest pore volume. Conversely, reducing the binder content clearly increases pore volume, with the 30M@C-25 sample exhibiting the highest value **(Figure S7b)**.

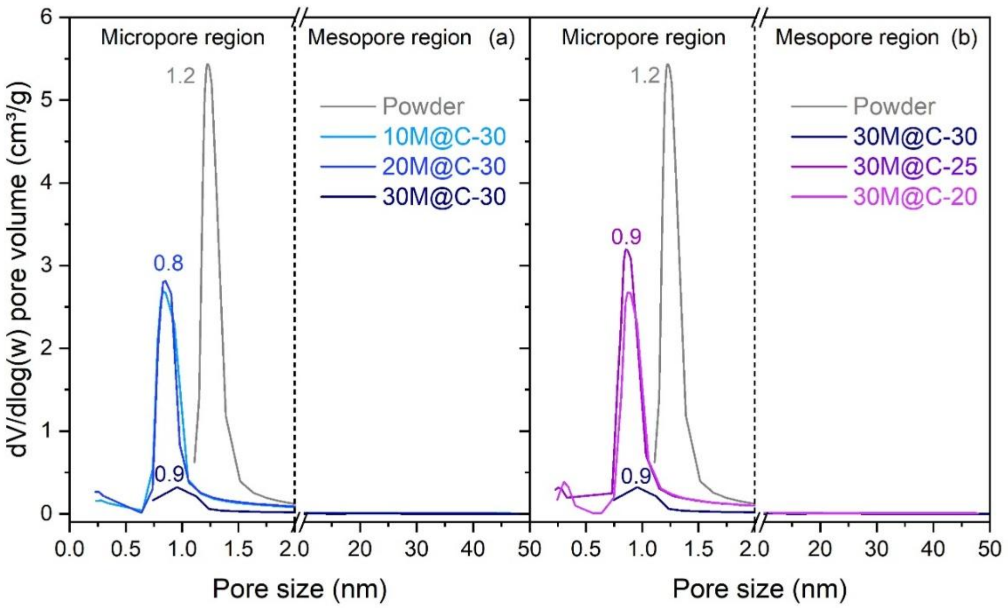

Figure S7. BJH pore size distribution curves of MOF-808 beads with (a) increasing solids fraction and (b) binder contents (30, 25 or 20 wt.%).

SEM images of MOF-808 powder along with surface views of MOF-808 beads with different solids fraction are displayed in **Figure S8**.

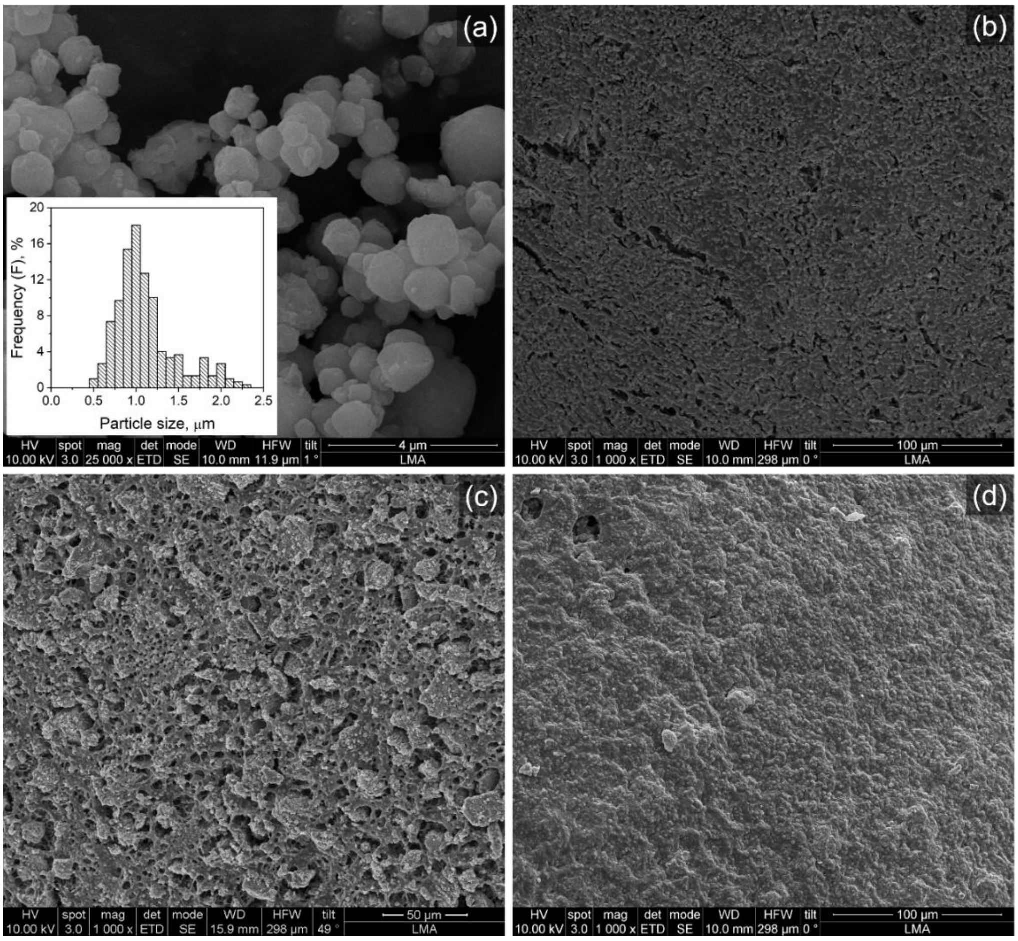

Figure S8. SEM micrographs of (a) MOF-808 powder together PSD histogram and SEM external surface views of (b) 10M@C-30, (c) 20M@C-30 and (d) 30M@C-30 MOF-808 beads.

As shown in **Figure S8a**, MOF-808 powder is formed by particles with octahedral morphology and with an average diameter of 1 ±0.29 µm, which present some extent of agglomeration. In general, it can be appreciated that the external surface (or shell) of beads is formed by MOF-808 particles entrapped in HPMC welted polymeric walls (**Figures S8b-d**). Noteworthy, porosity of external surface changes with solids loading of the initial suspension. In the case of 10M@C-30 beads (**Figure S8b**), the surface contains small and randomly distributed pores of around 1.6 µm and 4.3 µm, respectively. However, upon farther increasing solids loading of suspension to 30 vol.%, the surface porosity is dramatically reduced (see **Figure S8d**). In fact, 30M@C-30 bead seems to be covered by commercial methylcellulose shell, being barely possible to observe macropores by SEM (i.e., the presence of mesopores cannot be ruled out).

Figure S9 compares external (**Figures S9a and c**) as well as cross section (**Figures S9b and d**) views of 30M@C-30 and 30M@C-25 beads, respectively. Overall, 30M@C-25 beads exhibit a significantly more open porous structure with greater accessibility to MOF particles compared to the 30M@C-30 beads. In fact, unlike 30M@C-30 beads surface, the surface of 30M@C-25 beads presents randomly distributed pores with an average size of 1.97 µm (**Figure S9c**). Additionally, the cross section of 30M@C-25 contains a higher amount lamellar oriented pores (4.31 µm) (**Figure S9d**) rather than the larger, circular pores (12 µm) observed for 30M@C-30 beads (**Figure S9d**). These differences in pore morphology and size suggest that dendrites could form more effectively when using less binder.

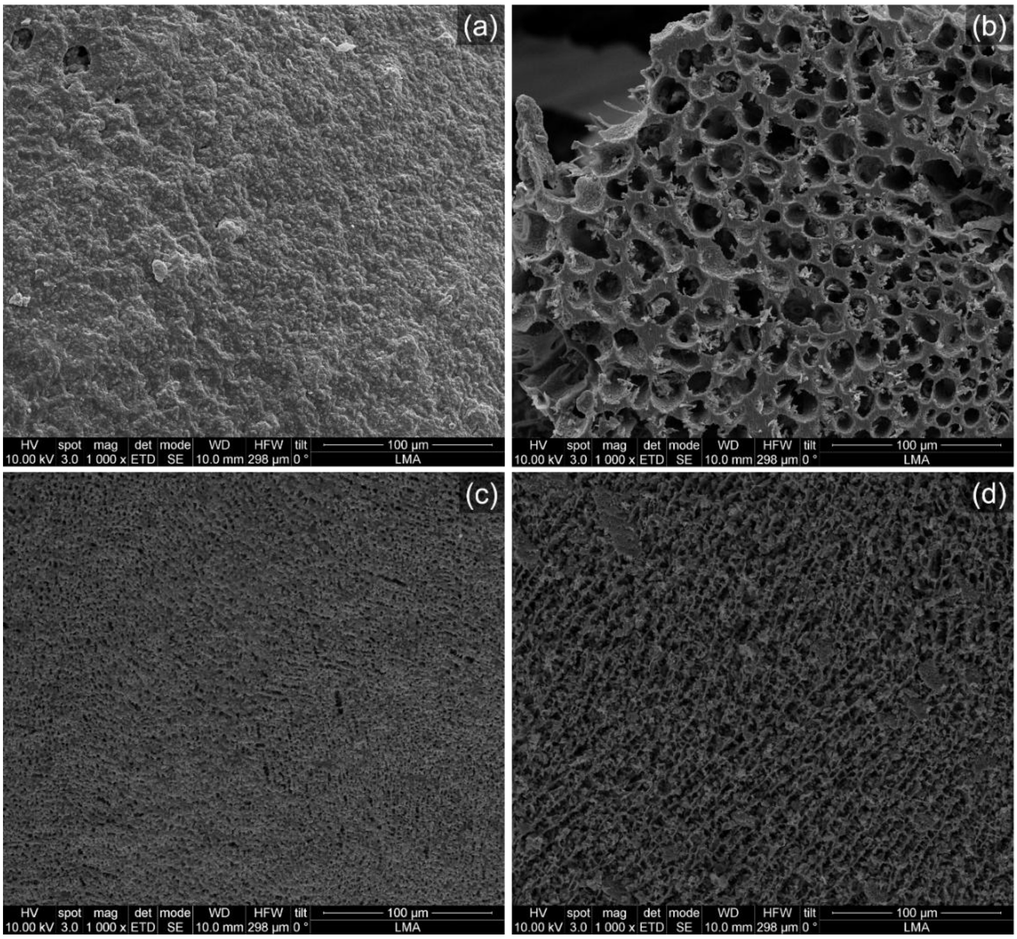

Figure S9. SEM micrographs of (a, c) external surface views and (b, d) cross section views of 30M@C-30 and 30M@C-25 beads, respectively.

S2. Shaping of UiO-66-NH_2 and MIL-100(Fe) by freeze spherification

S2.1. Experimental

S2.1.1. Synthesis of gel-based UiO-66-NH2 and MIL-100(Fe) powder

UiO-66-NH_2 gel was synthesized by first dissolving 0.788 g of $ZrCl_4$ in a 4:2 (v/v) mixture of DMF and water (total volume: 6 mL). Separately, 1.13 g of 2-aminoterephthalic acid was dissolved in 15 mL of DMF. After that, two solution were combined, forming a yellow mixture that was heated to 80 °C and maintained at this temperature for 1h to induce gelation. The resulting gel was subjected to a solvent exchange process to remove unreacted species, sequentially washing three times each with DMF, ethanol, and water. Finally, the purified gel was freeze-dried in a lyophilizer at -42°C for 8 hours, yielding a yellow powder.

On the other hand, MOF MIL-100(Fe) was synthesized using a hydrothermal method. First, 3.22 g of benzene-1,3,5-tricarboxylic acid (trimesic acid) was dissolved in 60 mL of Milli-Q water under vigorous stirring, followed by the gradual addition of 1.74 g of NaOH to aid dissolution. The mixture was stirred thoroughly until fully dissolved, with mild heating (up to 50°C) to facilitate dissolution. Separately, 4.08 g of $FeCl_2 \cdot 4H_2O$ was dissolved in 100 mL of Milli-Q water at room temperature under stirring. The trimesic acid solution was then added dropwise to the $FeCl_2$ solution under continuous stirring, and the reaction mixture was allowed to stand at room temperature for 24 hours. The resulting brown precipitate was collected by centrifugation at 3500 rpm for 5 minutes, followed by sequential washing steps to remove unreacted reagents. The solid was washed three times with water and three times with ethanol, each step involving resuspension and centrifugation. Finally, the purified MIL-100(Fe) was dried at room temperature for 24 hours.

S2.1.2. Shaping by freeze spherification

Gel-based UiO-66-NH_2 and MIL-100(Fe) beads where shaped by freeze spherification, as detailed in the Experimental section of the manuscript. The same composition was used for shaping UiO-66-NH_2 and MIL-100(Fe) beads, employing the optimal conditions consisting of suspension solids loading of 30 vol.% and 25 wt.% HPMC binder. Following the established nomenclature, the UiO-66-NH_2 and MIL-100(Fe) beads are denoted as 30U-NH_2@C-25 and 30M(Fe)@C-25, respectively.

S2.1.3. Characterization techniques

All samples, in both powder and bead form, were characterized by XRD, TGA, N_2physisorption and SEM, under the same operating conditions described in the Experimental section of the manuscript.

S2.2. Characterization results

Figure S10 shows XRD patterns of gel-based UiO-66-NH2 and MIL-100(Fe) in both powder and bead forms. The XRD pattern of UiO-66-NH_2 (**Figure S10a**) presents few and broad peaks at 7.3, 8.4 (shoulder) and 25.6 °2θ, which are characteristic of UiO-66-NH_2. The absence of other characteristic peaks suggests that gel-based UiO-66-NH_2 is a poorly crystalline solid. Note that the XRD pattern of 30U-NH_2@C-25 beads is very similar, indicating that crystallinity of MOF was not affected during shaping process. Instead, the XRD pattern of MIL-100(Fe) exhibits a higher number of sharp and well-defined peaks, which are characteristic of octahedral MIL-100(Fe)

crystals (200 nm). The significantly higher crystallinity of MIL-100(Fe) compared to UiO-66-NH_2 can be attributed to the synthesis method: the hydrothermal process yields a more highly ordered structure than gelation. In the case of 30M(Fe)@C-25 pattern (**Figure S10 b**), the same XRD peaks can be observed. Nevertheless, the relative intensity of peaks at lower angles are less intense for bead, which may suggest slight preferred orientation effects rather than significant loss of crystallinity.

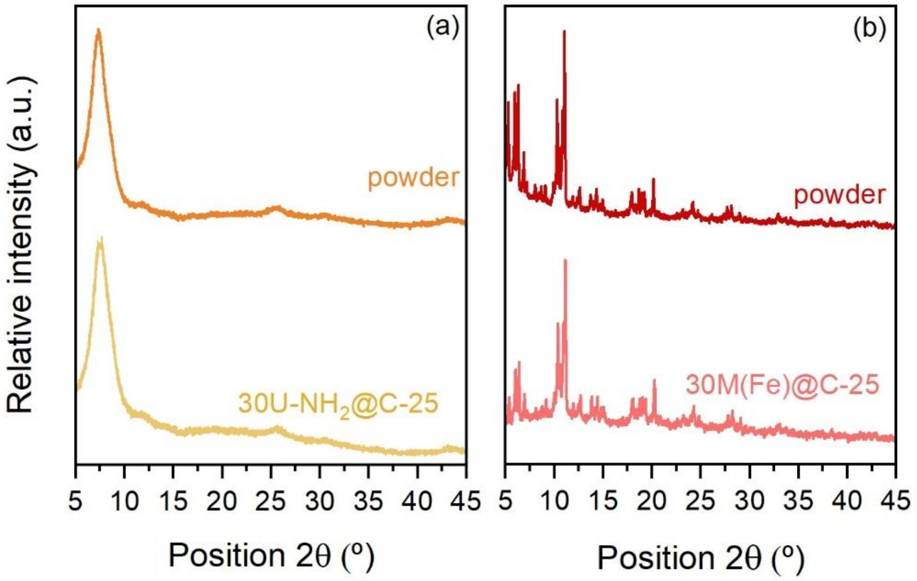

Figure S10. XRD spectra of (a) UiO-66-NH_2 and (b) MIL-100(Fe) powder and beads.

TGA profiles of UiO-66-NH_2 and MIL-100(Fe) samples are included in **Figure S11a** and **FigureS11b**, respectively.

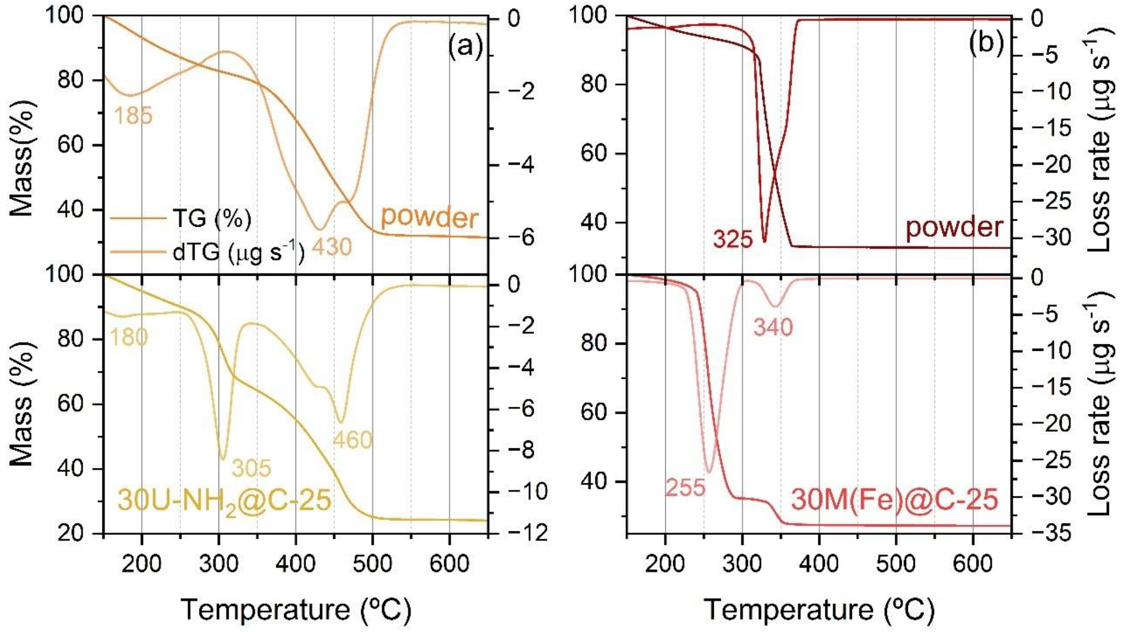

Figure S11. TG and dTG profiles of (a) UiO-66-NH_2 and (b) MIL-100(Fe) powder and beads.

The TG profile of UiO-66-NH_2 powder (**Figure S11a**) shows an initial continuous weight loss of around 20% between 150 and 350 °C, which is associated with removal of free solvent and ligand (NH_2 groups). Above 350 °C, mass loss rate increased due to UiO-66 structure decomposition, as revealed by the dTG curve. For 30U-NH_2@C-25 beads, the mass loss in the 150-350 °C range is higher (around 40 %), due to additional decomposition of HPMC at around 300 °C, as seen in the dTG profile. Beyond 350 °C, the mass loss profile is similar to that of powder, with complete decomposition occurring at around 500 °C. In contrast, MIL-100(Fe) barely losses mass up to 300 °C (**Figure S11b**), after which a drastic drop of mass is observed due to degradation of BTC ligand. Notably, the complete thermal degradation of MIL-100(Fe) beads starts at lower temperature (250 vs. 300 °C). In this case, a total mass loss of 73 wt.% is observed at 350 °resulting from the simultaneous degradation of linker and HPMC binder, obtaining 27 WT.% Fe_2O_3 as final residue.

Figure S12 displays N_2 adsorption-desorption isotherms of UiO-66-NH_2 and MIL-100(Fe) in both powder and beads form.

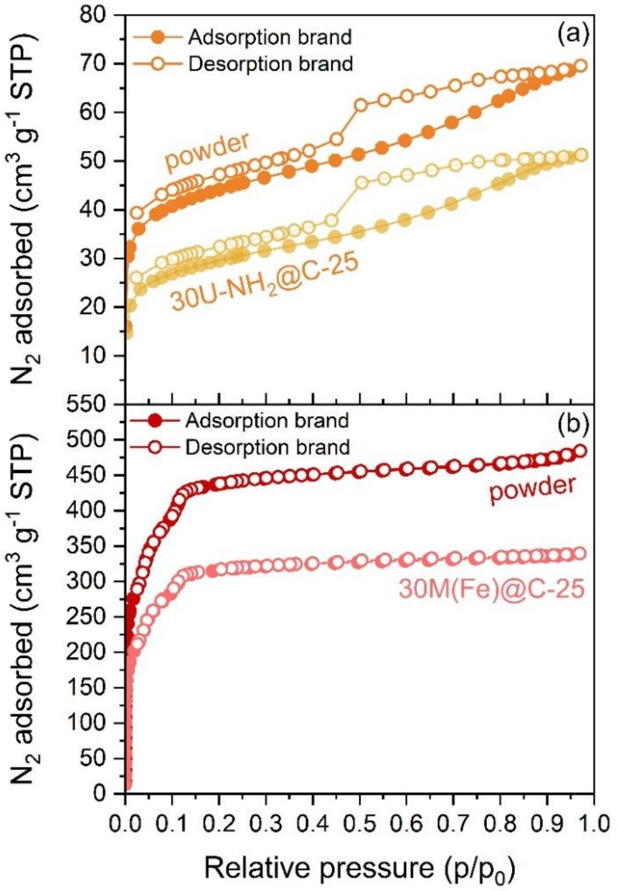

Figure S12. N_2 physisorption isotherms of (a) UiO-66-NH_2 and (b) MIL-100(Fe) samples.

The shape of the isotherms of gel-based UiO-66-NH_2 is type IV according to IUPAC, which is characteristic of mesoporous solid (**Figure S12a**). Indeed, the presence of hysteresis loop at around 0.5 relative pressure confirms the presence of mesopores with X shape. In contrast, MIL-100(Fe) powder physisorbs a notably higher amount of N_2 at lower relative pressures and its isotherm lacks and hysteresis loop instead exhibiting a plateau, indicative of a predominantly microporous structure (**Figure S12b**). Note that the different porosity arises from their different synthesis methods (gelation vs. hydrothermal). In both cases, the N_2 adsorption capacity of

beads and consequently their textural properties (summarized in Table S2) are lower than those of corresponding powder. This reduction is not only due to the introduction of non-porous solid (HPMC binder) but also due to some pore blockage caused by binder coating, as evidenced by normalized surface areas in **Table S2**. Notably, the surface accessibility or percentage of accessible surface area of 30U-NH_2@C-25 and 30M(Fe)@C-25 beads are 90 and 92%, respectively.

Table S2. Textural properties of UiO-66-NH_2 and MIL-100(Fe) powder and beads.

Sample	X_{HPMC} (wt.%)	*SSA* (m^2/g)	*NSSA* ($m^2\ g_{MOF}$)	Surf. acc. (%)	V_{pore} (cm^3/g)
UiO-66-NH_2	n.a.	152.3	152.3	n.a.	0.108
30U-NH2@C-25	23	103.1	133.9	88	0.088
MIL-100(Fe)	n.a.	1562.1	1562.1	n.a.	0.749
30M(Fe)@C-25	17	1115.0	1343.4	86	0.525

SEM images of both surface and cross-section views of both 30U-NH_2@C-25 and 30M(Fe)@C-25 beads are shown in **Figure S13**.

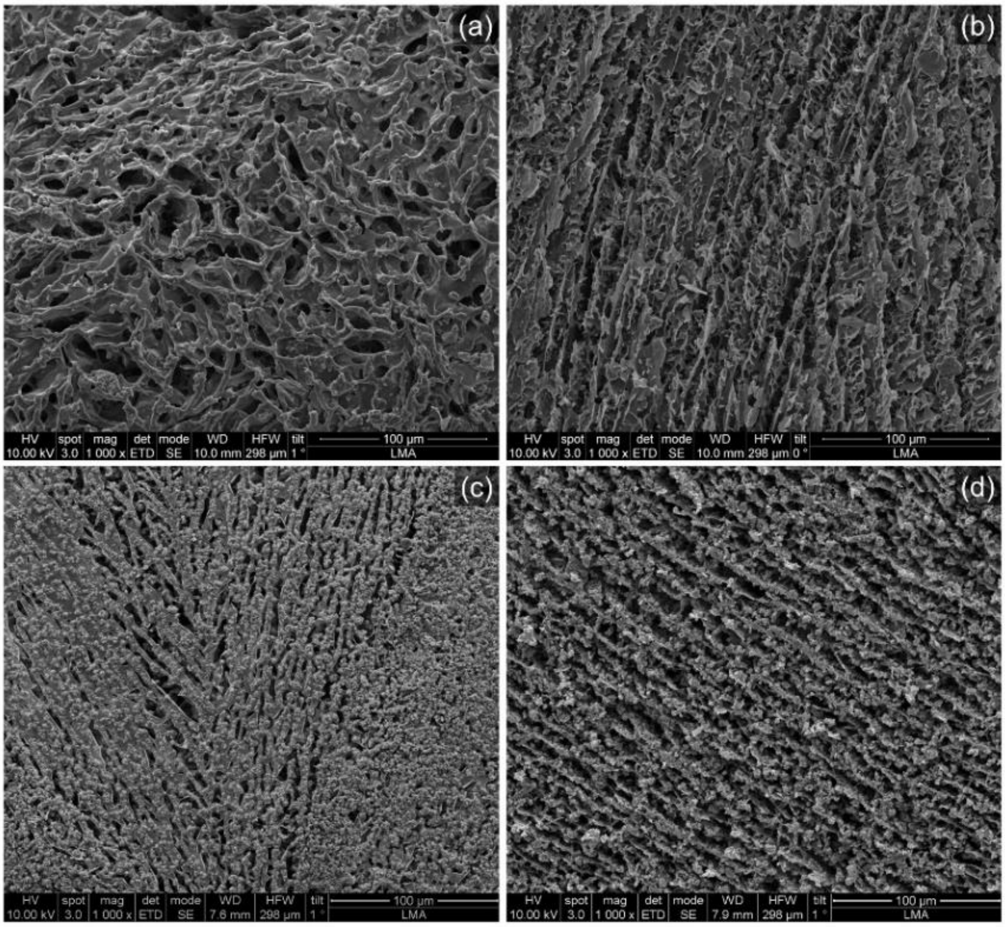

Figure S13. SEM micrographs of (a, c) external surface views and (b, d) cross section views of 30U-NH_2@C-25 and 30M(Fe)@C-25 beads, respectively.

As seen in **Figure S13a and c**, both beads exhibit macropores on their external surfaces, which have irregular shapes and sizes exceeding 3 µm. The external macropores of 30M(Fe)@C-25

appear more uniform. Notably, the internal porous structures of both 30U-NH_2@C-25 and 30M(Fe)@C-25 beads are similar, consisting of lamellar oriented macropores with wall-to-wall distances of around 12 and 7 µm, respectively (**Figures S13b and d**). Overall, the UiO-66-NH_2 and MIL-100(Fe) beads present surface accessibility and a porous structure comparable to that of 30M@C-25 beads, confirming that the optimized method is also effective in shaping other MOFs.
